# Transcriptomic analysis of the autophagy machinery in crustaceans

**DOI:** 10.1186/s12864-016-2996-4

**Published:** 2016-08-09

**Authors:** Saowaros Suwansa-ard, Wilairat Kankuan, Tipsuda Thongbuakaew, Jirawat Saetan, Napamanee Kornthong, Thanapong Kruangkum, Kanjana Khornchatri, Scott F. Cummins, Ciro Isidoro, Prasert Sobhon

**Affiliations:** 1 Department of Anatomy, Faculty of Science, Mahidol University, Bangkok, Thailand; 2 Department of Anatomy, Faculty of Science, Prince of Songkla University, Hatyai, Songkhla Thailand; 3 Chulabhorn International College of Medicine, Thammsat University, Klongluang, Phathumthani, Thailand; 4 Faculty of Science, Health, Education and Engineering, University of the Sunshine Coast, Maroochydore, QLD Australia; 5 Laboratory of Molecular Pathology, Department of Health Sciences, Amedeo Avogadro University, Novara, Italy; 6 Faculty of Allied Health Sciences, Burapha University, Chonburi, Thailand; 7 School of Medicine, Walailak University, Nakhon Si Thammarat, Thailand

**Keywords:** Autophagy, Crustaceans, Transcriptome, Nervous system, Ovary, Autophagy markers

## Abstract

**Background:**

The giant freshwater prawn, *Macrobrachium rosenbergii*, is a decapod crustacean that is commercially important as a food source. Farming of commercial crustaceans requires an efficient management strategy because the animals are easily subjected to stress and diseases during the culture. Autophagy, a stress response process, is well-documented and conserved in most animals, yet it is poorly studied in crustaceans.

**Results:**

In this study, we have performed an *in silico* search for transcripts encoding autophagy-related (Atg) proteins within various tissue transcriptomes of *M. rosenbergii*. Basic Local Alignment Search Tool (BLAST) search using previously known Atg proteins as queries revealed 41 transcripts encoding homologous *M. rosenbergii* Atg proteins. Among these Atg proteins, we selected commonly used autophagy markers, including Beclin 1, vacuolar protein sorting (Vps) 34, microtubule-associated proteins 1A/1B light chain 3B (MAP1LC3B), p62/sequestosome 1 (SQSTM1), and lysosomal-associated membrane protein 1 (Lamp-1) for further sequence analyses using comparative alignment and protein structural prediction. We found that crustacean autophagy marker proteins contain conserved motifs typical of other animal Atg proteins. Western blotting using commercial antibodies raised against human Atg marker proteins indicated their presence in various *M. rosenbergii* tissues, while immunohistochemistry localized Atg marker proteins within ovarian tissue, specifically late stage oocytes.

**Conclusions:**

This study demonstrates that the molecular components of autophagic process are conserved in crustaceans, which is comparable to autophagic process in mammals. Furthermore, it provides a foundation for further studies of autophagy in crustaceans that may lead to more understanding of the reproduction- and stress-related autophagy, which will enable the efficient aquaculture practices.

**Electronic supplementary material:**

The online version of this article (doi:10.1186/s12864-016-2996-4) contains supplementary material, which is available to authorized users.

## Background

Macroautophagy (autophagy) is a complex process involving degradation of cellular constituents to maintain organelle and protein homeostasis in response to various stresses, including genomic, oxidative, hypoxic, proteotoxic and metabolic stresses as well as starvation [1]. Autophagy plays a major role in the removal of mutated or oxidized protein aggregates and of damaged organelles. It is also involved in restoring nutritional and metabolic imbalances during starvation. Deficiency in the clearing and restoring function of autophagy leads to cell death, which ultimately contributes to growth retardation and various degenerative diseases, including neurodegenerative, muscular and cardiovascular diseases [2–5].

Autophagy is an evolutionary conserved mechanism ubiquitously present in all eukaryotic cells. It is driven and regulated by several proteins designated as autophagy-related (Atg) proteins (ATG in mammals). At least 32 Atg genes have so far been identified in the yeast, *Saccharomyces cerevisiae*, the species initially employed for genetic screening of autophagy [6]. Many Atg orthologs have subsequently been identified in higher eukaryotes, including nematodes (*Caenorhabditis elegans*) [7], arthropods (*Drosophila*) [8], mammals (mouse and human) [9], as well as in plants [10]. Furthermore, these Atg genes seem to be highly conserved among different metazoan phyla. Amongst these, phylum Arthropoda has the largest number of species and their members are widespread throughout both terrestrial and aquatic habitats. While autophagy in insects has been studied intensively [8, 11–15], the autophagic machinery in crustaceans is still unexplored. However, a few studies have shown the presence of autophagosome within the cells of the midgut in crustacean species by using electron microscopy and it has been suggested that autophagy might play an important role for midgut cell survival [16–19].

Many species of crustaceans, especially decapods (shrimps and crabs), are a commercially important food source. During captivity in farming, these animals can be subjected to many stresses that affect feeding, growth, reproduction, and diseases. Hence, management of stress, disease, and reproductive manipulation are crucial in farming. Since these processes are related to autophagy [20–22], an understanding of the autophagic process in crustaceans is important for improvement of crustacean aquaculture. To date, the genome of the water flea, *Daphnia pulex*, had been generated [23]. Further, the transcriptomes of several crustaceans, including the mud crab (*Scylla olivacea*) [24] and white shrimp (*Litopenaeus vannamei*) [25], have been provided in the online databases. Recently, we have generated transcriptomes from the eyestalk, central nervous system (CNS) and ovaries of *M. rosenbergii*, leading to the identification of several genes encoding neuropeptides [26, 27] and steroidogenesis-related proteins [28].

In this study, we identified Atg genes in the above-mentioned *M. rosenbergii* transcriptomes and then examined the presence of key Atg proteins in the tissues using Western blotting and immunohistochemistry. Furthermore, structural comparisons between human and *M. rosenbergii* marker proteins for autophagic process, including Beclin 1 (BECN1), vacuolar protein sorting (Vps) 34, microtubule-associated proteins 1A/1B light chain 3 (MAP1LC3 or LC3), p62/sequestosome 1 (SQSTM1) and lysosomal-associated membrane protein 1 (Lamp-1), were performed to show that crustacean Atg marker proteins have a similar structure, and possibly functional activity, to those of human. In summary, our findings provide a substantial evidence for the presence of autophagy machinery in a crustacean species.

## Results

### Gene mining of autophagy-related proteins in *M. rosenbergii* transcriptomes

An *in silico* search of Atg proteins in *M. rosenbergii* transcriptomes (the eyestalk, CNS, and ovary combined datasets) revealed 41 transcripts predicted to encode Atg proteins (Table 1). Atg proteins are categorized based on their functions related to the progression of autophagic process as determined in yeast and mammals [29]. The first category includes the proteins involved in formation of the autophagy interactome that initiates the process of autophagy; the second category consists of proteins that are responsible for Atg9-WIPI1 complex formation, which triggers membrane nucleation and phagophore formation; the third category includes proteins that are involved in Atg12 conjugation, which mediates phagophore elongation; the fourth category comprises of proteins involved in Atg8/LC3 conjugation, which controls cargo sequestration and autophagosome formation; and the last category includes the phosphatidylinositol 3-phosphate (PI3P)-related proteins with other proteins associated with autophagic activities. Of the 41 transcripts, 23 transcripts appear to be full-length as determined by the presence of an initiation methionine and a stop codon. The *M. rosenbergii* Atg predicted proteins together with best BLAST hit (at lowest e-value) is provided in Additional file 1.

**Table 1:** Atg transcripts present in *M. rosenbergii* transcriptomes in comparison with Atg genes of mammals and yeast

Autophagy core machinary	Mammals	Yeasts	*Number of hit transcript in M. rosenbergii transcriptomes*
ULK1 protein kinase complex	ULK1	ATG1	1 (same hit with ULK2)
	ATG13/APG13	ATG13	1
	FIP200	ATG17	1
	ATG101		1
Atg9-WIPI1 complex	ATG9A, B	ATG9	2
	WIPI-1,2,3,4 Vps34-beclin1 class III PI3-kinase complex	ATG18	4
	PIK3C3/VPS34	VPS34	1
	PIK3R4/VPS15	VPS15	3
	BECN1	ATG6 (VPS30)	1
	ATG14	ATG14	1
	UVRAG	VPS38	1
	Rubicon		1
	AMBRA1		1
Atg12 conjugation	ATG12	ATG12	1
	ATG5	ATG5	1
	ATG16L1,L2	ATG16	1
	ATG7	ATG7	1
	ATG10	ATG10	1
LC3/Atg8 conjugation	MAP1LC3B/LC3B	ATG8	1
	GABARAP	ATG8	1
	GATE-16	ATG8	1
	GABARAPL1	ATG8	1 (same hit with GABARAP)
	ATG7	ATG7	(see Atg7 above)
	ATG3	ATG3	1
	ATG4A-D (autophagins 1–4)	ATG4	3
PI(3)P-related proteins	ALFY/WDFY3		1
	DFCP1/ZFYVE1		1
	FYCO1		2
	MTMR14		2
	MTMR3		1
Others	ULK2		1
	Sequestosome-1(SQSTM-1)/p62		1
	beclin 1 associated autophagy related key regulator like		1
	ATG2		1

The number of predicted Atg transcripts derived from *M. rosenbergii* transcriptomes is indicated

### Sequence analyses of Atg marker proteins in *M. rosenbergii*

**Fig. 1:**
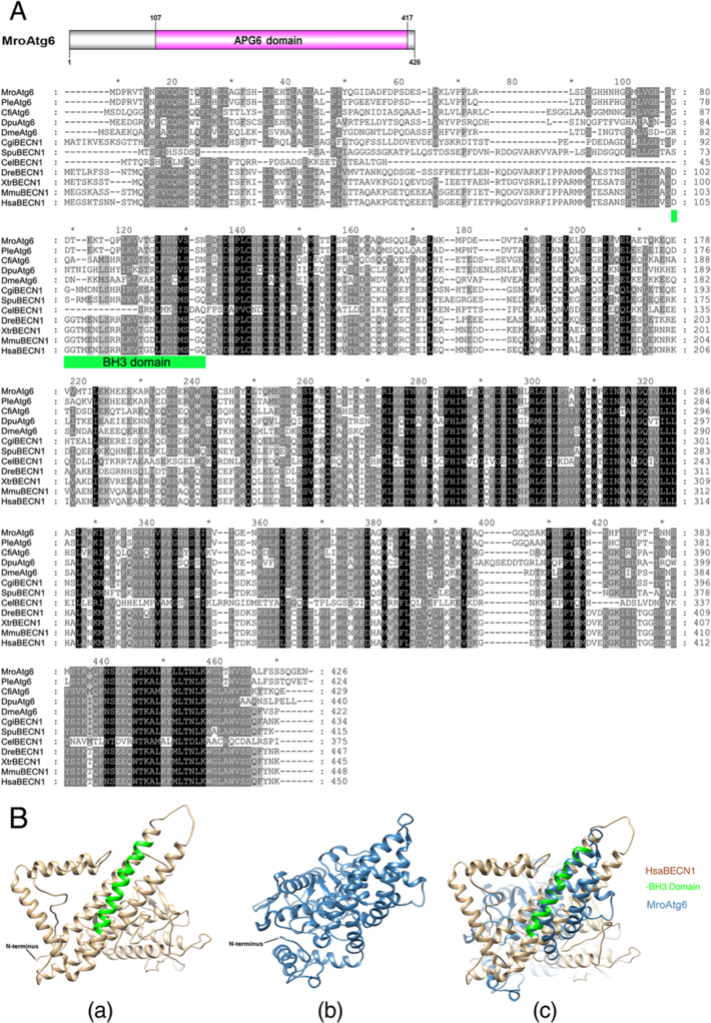
Identification of an *M. rosenbergii* Atg6. **a** A schematic annotation of *M. rosenbergii* Atg6 (MroAtg6) and the alignment of crustacean Atg6 proteins with Atg6/BECN1 of other species from different phyla. Black shading indicates conserved amino acids while grey shading indicates similar amino acids. Green bar indicates the region of Bcl-2 homology 3 (BH3) domain of HsaBECN1. Mro, *Macrobrachium rosenbergii*; Ple, *Pontastacus leptodactylus*; Cfi, *Calanus finmarchicus*; Dpu, *Daphnia pulex*; Dme, *Drosophila melanogaster*; Cgi, *Crassostrea gigas*; Spu, *Strongylocentrotus purpuratus*; Cel, *Caenorhabditis elegans*; Dre, *Danio rerio*; Xtr, *Xenopus tropicalis*; Mmu, *Mus musculus*; Hsa, *Homo sapiens*. **b** Predicted tertiary structures of HsaBECN1 (*a*) and MroAtg6 (*b*) and superimposition of HsaBECN1 and MroAtg6 (*c*). The BH3 domain is labeled on HsaBECN1, corresponding to amino acid positions 105–129

Further characterization, sequence alignment and structural comparisons of selected *M. rosenbergii* (Mro) Atg proteins that are considered to be important markers for monitoring the autophagic process (including Atg6/BECN1, Vps34, Atg8/LC3, and p62/SQSTM1), and for autophagosome-lysosome fusion (Lamp-1) [30, 31], were analyzed. The MroAtg6 consists of 426 amino acid residues in which the APG6 domain (Pfam accession number: PF04111) was annotated (position 107–417, Fig. 1a). Based on sequence alignment, the *M. rosenbergii* Atg6 protein shares 61–91 % similarity with Atg6 proteins of other crustacean species, while crustacean Atg6 proteins display 54–62 % similarity to the human ortholog, *Homo sapiens* (Hsa) BECN1 (which shows 85–98 % similarity with orthologs from other vertebrate species) (Fig. 1a). Conserved amino acids were observed throughout the entire length of the protein. Structural superimposition of HsaBECN1 and MroAtg6 revealed a similar conformation, including within the Bcl-2 homology 3 (BH3) domain that corresponds to amino acid positions 105–129 of HsaBECN1 (Fig. 1b).

**Fig. 2:**
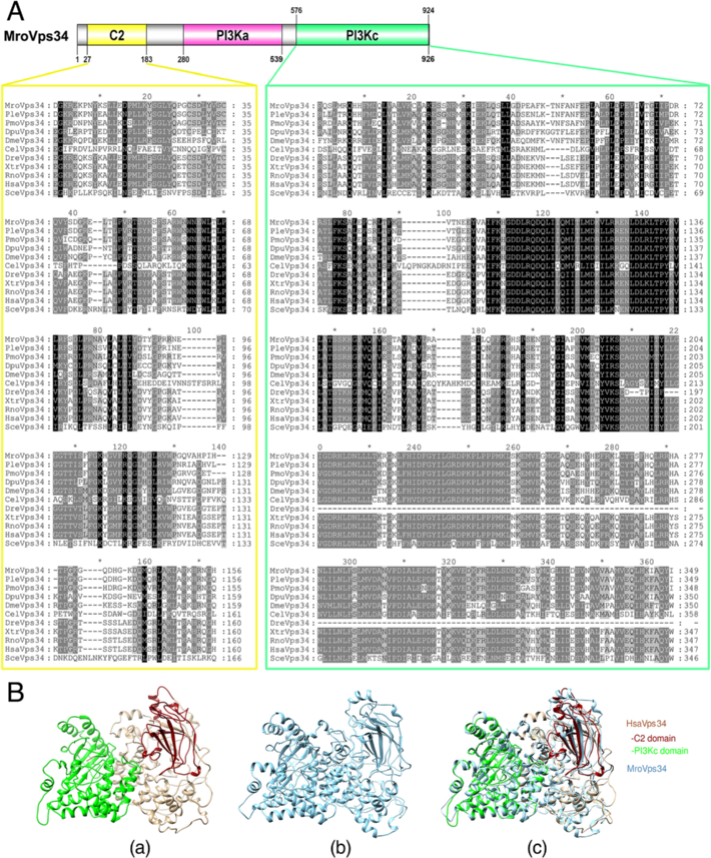
Identification of an *M. rosenbergii* Vps34. **a** A schematic annotation of *M. rosenbergii* Vps34 (MroVps34) and the alignment of Vps34 in crustaceans and other species from different phyla (only the alignments of amino acids within C2 and phosphatidylinositol 3-kinase catalytic (PI3Kc) domains are shown). Black shading indicates conserved amino acids while grey shading indicates similar amino acids. Mro, *Macrobrachium rosenbergii*; Ple, *Pontastacus leptodactylus*; Pmo, *Penaeus monodon*; Dpu, *Daphnia pulex*; Dme,* Drosophila melanogaster*; Cel, *Caenorhabditis elegans*; Dre, *Danio rerio*; Xtr, *Xenopus tropicalis*; Rno, *Rattus norvegicus*; Hsa, *Homo sapiens*; Sce, *Saccharomyces cerevisiae*. **b** Predicted tertiary structures of HsaVps34 (*a*) and MroVps34 (*b*) and superimposition of HsaVps34 and MroVps34 (*c*). The C2 (amino acid positions 26–186) and PI3Kc (amino acid positions 539–885) domains of HsaVps34 are labeled in red and green colors, respectively

Global protein sequence alignment showed that MroVps34 shares 65–90 % similarity with Vps34 proteins of other crustacean species, while crustacean Vps34 proteins share ~65–76 % similarity to the human ortholog (HsaVps34). Conserved functional domains, including C2 (positions 27–183; Pfam accession number: PF00792), phosphatidylinositol 3-kinase (PI3K) accessory (PI3Ka; positions 280–539; Pfam accession number: PF00613), and PI3K catalytic (PI3Kc; positions 576–924; Pfam accession number: PF00454) domains could be annotated in MroVps34 (Fig. 2a). A high degree of amino acid conservation was found throughout the protein length, especially within C2 and PI3Kc domains (Fig. 2a). Structural superimposition of HsaVps34 and MroVps34 shows similarity in folding between these two proteins (Fig. 2b).

**Fig. 3:**
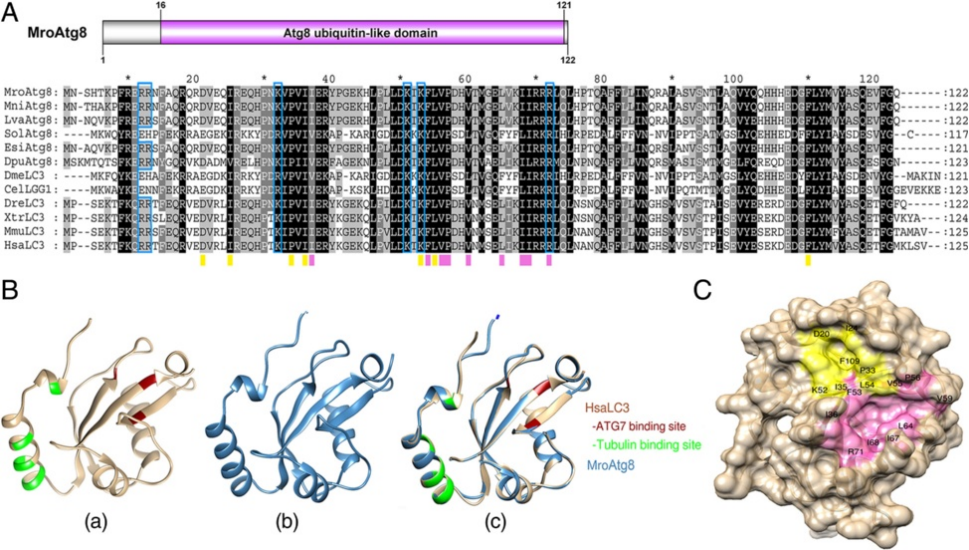
Identification of an *M. rosenbergii* Atg8. **a** A schematic annotation of *M. rosenbergii* Atg8 (MroAtg8) and the alignment of crustacean Atg8 proteins with Atg8/LC3 of other species from different phyla. Black shading indicates conserved amino acids while grey shading indicates similar amino acids. Blue boxes indicate the basic residues which are previously known to be critical for electrostatic interaction of LC3 with other proteins. Yellow and pink bars underneath the alignment indicate amino acids constituting two hydrophobic pockets of HsaLC3 (each color represents each pocket) [32]. Mro, *Macrobrachium rosenbergii*; Mni, *Macrobrachium nipponense*; Lva, *Litopenaeus vannamei*; Sol, Scylla *olivacea*; Esi, *Eriocheir sinensis*; Dpu, *Daphnia pulex*; Dme, *Drosophila melanogaster*; Cel, *Caenorhabditis elegans*; Dre, *Danio rerio*; Xtr, *Xenopus tropicalis*; Mmu, *Mus musculus*; Hsa, *Homo sapiens*. **b** Predicted tertiary structures of HsaLC3 (*a*) and MroAtg8 (*b*) and superimposition of HsaLC3 and MroAtg8 (*c*). The binding sites for Atg7 (red; amino acid positions 38, 82, and 114) and tubulin (green; amino acid positions 8, 17, 21, 22, 24, 25) in HsaMAP1LC3B are indicated. **c** Predicted MroAtg8 tertiary structure with hydrophobic surface. Residues which were predicted to form two hydrophobic pockets in MroAtg8 are colored in yellow and pink

The deduced MroAtg8 protein and homologs from *Macrobrachium nipponense*, *Litopenaeus vannamei*, *Scylla olivacea*, *Eriocheir sinensis*, and *D. pulex*, were identified. Alignment of the crustacean MroAtg8 with those of non-crustacean species indicates a highly conserved primary amino acid sequence (average similarity ~60 %, Fig. 3a). The MroAtg8 consists of 122 amino acids, with 98 % identity to *M. nipponense* Atg8. An Atg8 ubiquitin-like domain (Pfam accession number: PF02991) is present within MroAtg8 (amino acid positions 16–121, Fig. 3a). The MroAtg8 and human MAP1LC3B (HsaMAP1LC3B) share 72 % similarity, and their structural superimposition indicates a similar secondary structure, including at the binding sites for Atg7 and tubulin, as shown in HsaMAP1LC3B (Fig. 3b). The amino acid residues which are responsible for the interaction between Atg8/MAP1LC3B and other autophagy receptors [32], and the amino acid residues which are important for the formation of two binding pockets are shown in Fig. 3a. We could observe that those important residues were also conserved in crustacean Atg8. Therefore, the basic residues important for electrostatic interaction of MroAtg8 with other proteins could be predicted, possibly including R_11_, R_12_, K_31_, K_50_, K_52_, and R_71_ (Fig. 3a). Likewise, the residues required for the formation of two binding pockets in MroAtg8 could be predicted (D_20_, I_24_, P_33_, I_35_, K_52_, L_54_, and F_109_ for the formation of the first pocket and I_36_, F_53_, V_55_, P_56_, V_59_, L_64_, I_67_, I_68_, and R_71_ for the formation of the second pocket) and also mapped in the predicted MroAtg8 tertiary structure (yellow and pink areas, respectively; Fig. 3c), assembling the appearance of two adjacent pockets.

**Fig. 4:**
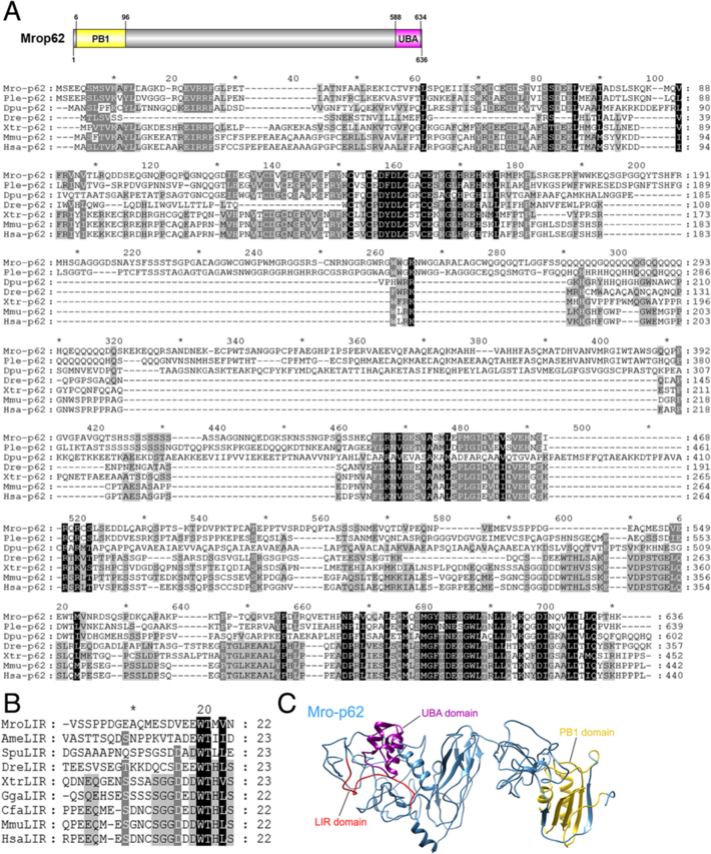
Identification of an *M. rosenbergii* p62. **a** A schematic annotation of *M. rosenbergii* p62 (Mrop62) and the sequence alignment of crustacean p62 proteins with p62 of other species from different phyla. Black shading indicates conserved amino acids while grey shading indicates similar amino acids. Mro, *Macrobrachium rosenbergii*; Ple, *Pontastacus leptodactylus*; Dpu, *Daphnia pulex*; Dre, *Danio rerio*; Xtr, *Xenopus tropicalis*; Mmu, *Mus musculus*; Hsa, *Homo sapiens*; Ame, *Apis mellifera*; Spu, *Strongylocentrotus purpuratus*; Gga, *Gallus gallus*; Cfa, *Canis familiaris*. **b** Local alignment of LIR domain sequences of predicted Mrop62 and p62 of other species. **c** Predicted tertiary structure of Mrop62. The predicted Phox and Bem1p (PB1; amino acid positions 6–96), ubiquitin-associated (UBA; amino acid positions 588–634), and LC3-interacting region (LIR; amino acid positions 534–555) domains of Mrop62 are colored in yellow, purple, and red, respectively

Mrop62 is composed of 636 amino acids in which two conserved domains, the Phox and Bem1p (PB1; positions 6–96; Pfam accession number: PF00564) and ubiquitin-associated (UBA; positions 588–634; Pfam accession number: PF16577) domains, were annotated (Fig. 4a). Alignment of crustacean p62/SQSTM1 with those of vertebrates indicates a significant difference in primary amino acid composition throughout the length of protein, as well as several gaps (Fig. 4a). However, conservation was evident within the C-terminal UBA domain, e.g. amino acid positions 394–429 for Hsap62 [33, 34]. Based on previously known LC3 interacting region (LIR) of p62 in other animals [35] and the local alignment of LIR (Fig. 4b), we could predict the LIR region of Mrop62 which may comprise of amino acid residues at positions 534–555 (Fig. 4b and c). Tertiary structure comparison between Hsap62 and Mrop62 was not established because of a vast difference in primary amino acid composition between these two proteins.

**Fig. 5:**
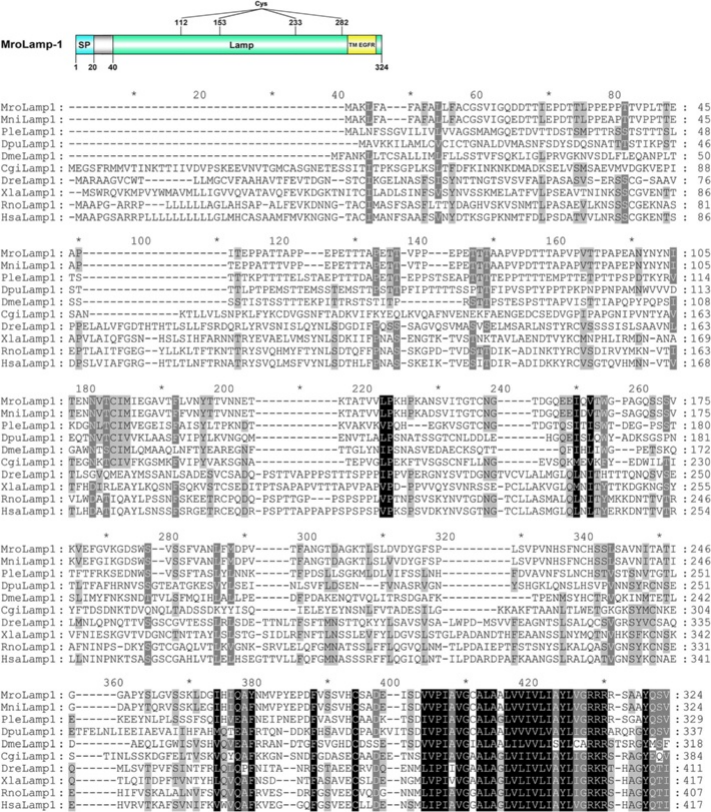
Identification of an *M. rosenbergii* Lamp-1. A schematic annotation of *M. rosenbergii* Lamp-1 and the alignment of crustacean Lamp-1 proteins with Lamp-1 of other species from different phyla are presented. A signal peptide (blue), a mature peptide (green), a conserved transmembrane domain of the epidermal growth factor receptor (TM-EGFR) (yellow), and cysteine residues are indicated. Black shading indicates conserved amino acids while grey shading indicates similar amino acids. Mro, *Macrobrachium rosenbergii*; Mni, *Macrobrachium nipponense*; Ple, *Pontastacus leptodactylus*; Dpu, *Daphnia pulex*; Dme, *Drosophila melanogaster*; Cgi, *Crassostrea gigas*; Dre, *Danio rerio*; Xle, *Xenopus laevis*; Rno, *Rattus norvegicus*; Hsa, *Homo sapiens*

Lamp-1 transcripts could be identified within crustacean sequence databases (*M. nipponense*, *P. leptodactylus*, and *D. pulex*) as well as in *M. rosenbergii* transcriptomes. Global sequence alignment indicates a variable amino acid composition among different species (19–95 % similarity), but a conservation at the C-terminal region corresponding to a transmembrane domain of the epidermal growth factor receptor (TM-EGFR) was observed (Fig. 5). Annotation of MroLamp-1 indicates a signal peptide (position 1–20) and a Lamp-1 mature protein (position 21–324) (Fig. 5a). The MroLamp-1 mature protein contains four cysteine residues (positions 112, 153, 233, and 282), a Lamp domain (position 40–324; Pfam accession number: PF01299), and a TM-EGFR domain (position 288–318; Pfam accession number: PR00316) (Fig. 5). However, the secondary structure of mature MroLamp-1 (as soluble form) was not predicted because Lamp-1 is a transmembrane protein [36].

### Spatial expression of autophagy markers

**Fig. 6:**
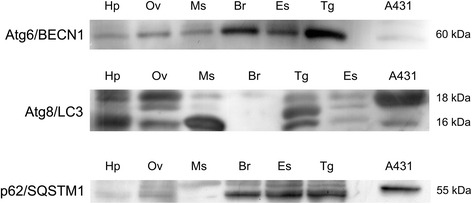
Validation of the autophagy marker proteins in female *M. rosenbergii* tissues. Western blotting shows the expression of Atg6/BECN1, Atg8/LC3, and p62/SQSTM1 in various tissues, including the hepatopancreas (Hp), ovary (Ov), muscle (Ms), brain (Br), eyestalk (Es), and thoracic ganglia (Tg). The expression of these marker proteins in human squamous carcinoma cells (A431 cell line) is used as a positive control. The amount of total protein loaded per lane was 30 μg

We examined the presence of corresponding Atg proteins in the female prawn by Western blotting and immunohistochemistry. Since there were no commercial antibodies available for *M. rosenbergii* Atg proteins, we used antibodies raised against the human Atg proteins, including Atg6/BECN1, Atg8/LC3, p62/SQSTM1 and Lamp-1. Western blotting indicated the presence of Atg6/BECN1, Atg8/LC3 and p62/SQSTM1 in various tissues, including the hepatopancreas, ovary, muscle, brain, eyestalk, and thoracic ganglia (Fig. 6). Based on band intensity, Atg6/BECN1 and p62/SQSTM1 were abundant in the nervous tissues (eyestalk, brain, and thoracic ganglia) and less abundant in other tissues such as the hepatopancreas, ovary, and muscle (Fig. 6). The Atg8/LC3 protein was detected prominently in the hepatopancreas, ovary, muscle, and thoracic ganglia, while its expression with less intensity was detected in the eyestalk (Fig. 6).

**Fig. 7:**
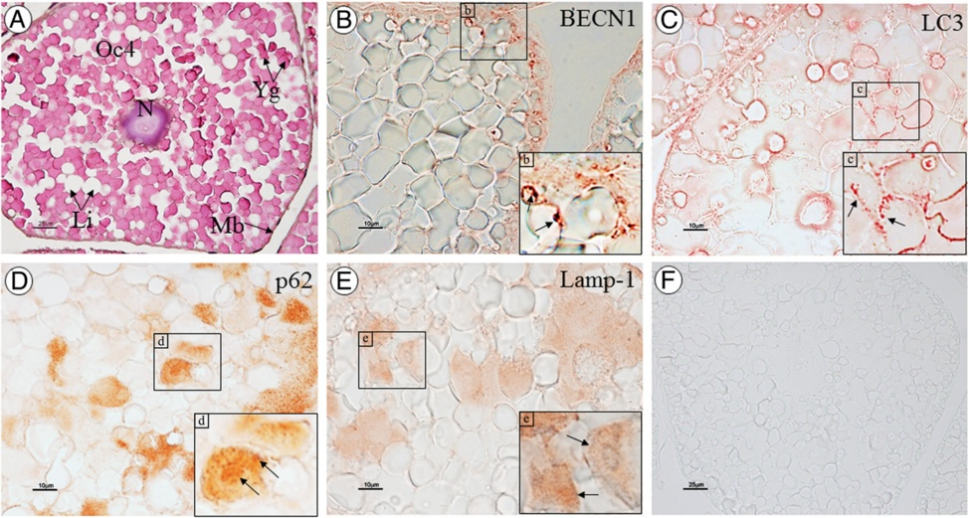
Immunoperoxidase staining of Atg marker proteins in the ovary of *M. rosenbergii*. Late vitellogenic oocyte (Oc4) with H&E stain (**A**). Immunolocalization of Atg6/BECN1 (**B**), Atg8/LC3 (**C**), p62/SQSTM1 (**D**), and Lamp-1 (**E**) in the Oc4 of ovarian sections. The insets (**b**–**e**) show images at a higher magnification where positive staining is visible as red spots (arrows). **F** Negative control. Scale bars: 25 μm (**A** and **F**) and 10 μm (**B**–**E**). (N, nucleus; Yg, yolk granules; Li, lipid droplets; Mb, cell membrane)

By immunoperoxidase staining, Atg6/BECN1, Atg8/LC3, and p62/SQSTM1 immunoreactivities were prominently detected in the cytoplasm of the vitellogenic oocytes (oocyte stages 3 and 4; Fig. 7A–D), while these proteins were less evident in the oogonia, previtellogenic oocytes (oocytes 1 and 2), and follicular cells (data not shown). In addition, expression of Lamp-1 in the ovarian tissue was investigated and its immunoreactivity showed a pattern similar to that of other autophagy marker proteins (Fig. 7E). No immunoreactivity was observed in the negative control (Fig. 7F).

## Discussion

Autophagy refers to a lysosome-driven process for the degradation of cellular components that are redundant, aged, or damaged [37]. The control and execution of autophagy have been well characterized in the yeast *S. cerevisiae* [38] and the worm *C. elegans* [39], and are being investigated in human, mouse, the fruit fly (*Drosophila melanogaster*), and plants. Here we present the first study showing that the key genes and proteins involved in the regulation and execution of autophagy are present in crustaceans.

All Atg proteins investigated could be identified from *M. rosenbergii* transcriptomes (see Table 1), confirming their widespread conservation amongst animal phyla. It is likely that the autophagic process in crustaceans follows the same pathway as in other animals, engaging a similar set of proteins from the initial triggering step (the activation of the ULK1 protein kinase complex) until the formation of active autophagic vesicles (the autolysosomes). The same hit transcripts were observed for GABARAP and GABARAPL1, and ULK-1 and −2 transcripts. In the case of GABARAP and GABARAPL1, it has been shown that there are at least three human *GABARAPLs* (1–3) genes which are homologues of the *GABARAP* gene [40]. Therefore, GABARAP and GABAAPL1 share a high sequence similarity and both, as well as other GABARAPLs, are categorized within the GABARAP family [41]. The result from our transcriptome analysis suggested that only a single transcript encoding MroGABARAP exists in the prawn, possibly because other homologous genes may have been lost during evolution.

Atg6 (Vps30 in yeast) was initially isolated as an interactor of the anti-apoptotic protein Bcl-2, and thereafter named Beclin 1 [42]. Atg6/BECN1 was next shown to be an essential activator of Vps34 for the initiation of the autophagic process [43]. High conservation of protein amino acid sequence, especially at the C-terminal region, could be observed in this protein among vertebrates and invertebrates, indicating that those conserved motifs are significant for the activity of this protein. In particular, amino acid conservation within the BH3 domain region and structural superimposition of human BECN1 and *M. rosenbergii* Atg6 indicates that *M. rosenbergii* and other crustacean Atg6 may interact with Bcl-2, and thereby play a regulatory role in apoptosis. Upon interaction with BECN1, the PI3K class III protein (PI3KC3 in human or Vps34 in yeast) catalyzes the formation of PI3P, which is essential for the recruitment of autophagosomal membranes [44, 45]. Vps34 protein is the most evolutionarily conserved protein within the PI3 kinase family [46, 47]. We did consistently find that crustacean Vps34 showed high conservation in their amino acid sequences with those of other invertebrates and vertebrates, especially at the domains described as critical for function. In fact, the Vps34 protein contains three major domains, namely the C2, PI3Ka, and PI3Kc domains [47], which were present in the deduced MroVps34. Vps34/PI3KC3 is known to interact with other autophagic proteins such as Atg14 and UV radiation resistance-associated gene (UVRAG) [48, 49]. Since Atg14 and UVRAG proteins were found in *M. rosenbergii* transcriptomes (Table 1) and the predicted structural conformation of *M. rosenbergii* Vps34 is similar to human Vps34 (Fig. 2b), we suggest that MroVps34 may exhibit a similar catalytic activity and bind to Atg14 and UVRAG.

Atg8/MAP1LC3B is a protein that is inserted into the nascent autophagosomal membranes upon post-translational lipidation of the microtubule-associated precursor [50]. Both length and amino acid composition were found to be similar among representative Atg8/MAP1LC3 sequences from different animal species (Fig. 3). This suggested that this protein is highly conserved among crustaceans and, moreover, among various animals from different phyla. Consistent with this, the predicted structural conformation of *M. rosenbergii* Atg8 exhibits a similar secondary structure to that of human MAP1LC3B, including within the regions that are known to be important for binding with Atg7 and tubulin proteins [51–54]. Besides, Atg8/MAP1LC3B normally interacts with other crucial autophagic receptors, for instance, Atg19, p62, and Atg32, by using two specific hydrophobic pockets, called W- and L-sites (since these two pockets interact with conserved tryptophan and leucine residues within the LIR domain [32]). We have shown that the amino acid residues known to be important for MAP1LC3B interaction with other proteins are preserved in crustacean Atg8, suggesting that they are critical for Atg8/MAP1LC3B functioning not only in vertebrates but also in crustaceans.

p62/SQSTM1 is a ubiquitin-binding protein that bridges the autophagy substrate to LC3 through its LIR domain [55]. Three distinct domains significant for p62 activity include a PB1 domain located at the N-terminus that is responsible for p62 self-assembly [56, 57]; a UBA domain located at the C-terminus that is important for p62-ubiquitin interacting [33, 58]; and a LIR domain that provides an interaction site for binding with Atg8/MAP1LC3B [55]. We found that p62 in crustaceans are not well conserved with other vertebrate p62, both in their length and amino acid composition. A large amino acid insertion could be observed within the middle region of the crustacean p62 when compared to vertebrate p62 sequences. In regard to the LIR motif, this region is highly conserved among vertebrate species but less conservation could be observed in invertebrate p62 proteins (Fig. 4b). However, we speculate that the important amino acids ‘WXXL’ (where X is any amino acid) within LIR, which binds directly to MAP1LC3B in mammals [32], are still preserved in invertebrate p62, including *M. rosenbergii*, with an exception for the leucine residue which is substituted by other aliphatic amino acids such as valine or isoleucine. Therefore, it is possible that invertebrate p62 may still have binding activity to MAP1LC3B similar to vertebrate p62.

Lamp-1 is an integral membrane protein of the endosomes and lysosomes, which is involved in the fusion of these organelles with autophagosomal vacuoles [31]. Despite low similarity in amino acid composition, we found that the MroLamp-1 preserves basic characteristics of Lamp-1 [59], including a conserved luminal Lamp domain (namely, the C-terminal TM-EGFR domain that extends to a short cytoplasmic tail). While other vertebrate Lamp-1 contains eight cysteine residues within the luminal region, hence forming four disulfide bonds [59], MroLamp-1 luminal domain contains only four cysteine residues potentially forming two disulfide bonds, and therefore likely not showing the looped-luminal domain with free-moving hinge as present in vertebrate Lamp-1 [59]. Considering a cytoplasmic tail of Lamp-1, this region contains the tyrosine-based motif (Y-X-X-I, where X is a polar residue) important for targeting Lamp-1 to lysosomes [60–62]. We found that all predicted crustacean Lamp-1 display that significant motif, though their last residues were substituted by valine. Although the intracellular targeting of Lamp-1 was conserved among crustaceans and other animals, a variable amino acid sequence within the N-terminal part and distinct Lamp domain characteristics suggests a different functional activity of crustacean Lamp-1 from those of vertebrates.

Based on sequence alignment, we found that the sequences of *M. rosenbergii* and human proteins at the regions used for producing anti-BECN1, anti-LC3, anti-p62 and anti-Lamp-1 antibodies shared 58.82, 42.86, 41.18 and 23.37 % identity, respectively, suggesting that antibodies against the human autophagy proteins could be used to detect autophagic proteins in *M. rosenbergii*. We therefore employed immunoblotting and immunocytochemistry techniques to validate the expression and subcellular localization of Atg marker proteins in *M. rosenbergii* tissues.

Autophagy has been shown to be present in the epithelial cells of the hepatopancreas and/or intestine in different crustacean species, including *Astacus astacus*, *Eubranchipus grubii* and *Neocaridina heteropoda*, in which autophagic activity was suggested to be essential for digestive cell survival (by eliminating degenerated organelles such as mitochondria) and a proper function of the digestive organs [16, 18, 19]. In our study, we found expression of Atg6/BECN1, Atg8/LC3, p62/SQSTM1 proteins to be widespread in various tissues of *M. rosenbergii* that were investigated (Fig. 6). Regarding Atg8/LC3 expression, there were three immunoreactive bands detected in prawn tissues. The largest (18 kDa) and smallest (16 kDa) bands may be considered as the LC3-I and-II forms, as compared with the LC3 expression in the human cell sample (Fig. 6). The middle band, which is present at an apparent molecular weight of 17.5 kDa in some tissue samples, probably represents the intermediate LC3-I that precedes the lapidated form of 16 kDa LC3-II [63]. Therefore, we suggest that the autophagic process is general to various prawn tissues, although the specific function of this process within different tissues still requires further investigation. In the ovary, we could observe that the levels of Atg6/BECN1, Atg8/LC3, p62/SQSTM1 and Lamp-1 expression within the cytoplasm increased in correlation with oocyte maturation, while no immunoreactivity was found in the follicular cells. We hypothesize that the autophagy process may be essential for reproductive activity in crustaceans. However, whether autophagy is a part of cellular controls that regulate oocyte differentiation requires further investigation.

## Conclusions

The present data supports the high evolutionary conservation of autophagic components in the crustacean lineage, as implicated by the existence of several key autophagy-related genes and proteins, and a high degree of sequence conservation of crucial autophagy marker proteins among crustaceans and other representative species from different phyla. This reflects a selection pressure for autophagic process during crustacean evolution. In addition, we have demonstrated that antibodies raised against human autophagy marker proteins can be used for monitoring the autophagic process in a crustacean model. Learning how autophagy is regulated in the giant freshwater prawn could open up new avenues for novel management in limiting infections, organ stress, as well as nutritional and metabolic imbalances due to feeding and starvation, which can ultimately impact on crustacean aquaculture.

## Methods

### Animals

Adult females *M. rosenbergii* were obtained from a local commercial farm in Ayutthaya province, Thailand. The prawns were anesthetized by immersion in ice water, and then the ovaries, hepatopancreases, muscles, brains, eyestalks, and thoracic ganglia were dissected out. Fresh tissues were kept at −80 °C for further use in transcriptome production and Western blotting (described below). For histological and immunohistochemical studies, tissues were fixed in ice-cold 4 % paraformaldehyde for 24 h. After washing with 70 % ethanol five times, specimens were then dehydrated in increasing concentrations of ethanol, cleared in xylene and infiltrated with paraffin, using an automated tissue processor. The paraffin-embedded ovarian tissue blocks were cut at 5 μm thickness and prepared for immunofluorescence staining.

### Transcriptome production and de novo assemblies of CNS and ovary of *M. rosenbergii*

Transcriptome data of *M. rosenbergii* was obtained from a previous study [26]. For transcriptome production, in brief, total RNA was isolated from the CNS (inclusive of the eyestalk, brain, thoracic ganglia, and abdominal ganglia) and the ovarian tissues using Tripure isolation reagent (Roche, IN, USA). The quality of total RNA of each tissue was checked by gel electrophoresis and spectrophotometry (NanoDrop 1000; Thermo Fisher Scientific, DE, USA). Twenty micrograms of total RNA of each tissue was sent to BGI, Hong Kong, for library construction. Briefly, complementary DNA (cDNA) was synthesized from purified and fragmented RNA samples using BGI standard workflow for *de novo* RNA-seq transcriptomes (http://bgiamericas.com/). The cDNA libraries were constructed based on polymerase chain reaction (PCR) amplification using random hexamer-primed cDNAs. Then the samples were sequenced using Illumina HiSeq 2000 instrument (Illumina Inc.) and only clean reads were used for further sequence assembly. De novo assembly was generated by SOAPdenovo software [64] using a combined CNS and ovary dataset with parameter set as follows: seqType, fq; minimum kmer coverage = 4; minimum contig length of 100 bp; group pair distance = 250. All sequence data of *M. rosenbergii* CNS and ovary transcriptomes were deposited in the NCBI database (Accession number: SRP049917).

### Gene mining, characterization, and sequence alignment

Searching of Atg proteins in *M. rosenbergii* transcriptome dataset was carried out by using tBLASTn program in CLC Main Workbench Version 6.0 (CLC Bio-Qaigen, AsiaPac, Taiwan) for which known Atg proteins (from mammals, yeast, and insects) were used as queries. The parameter was set as follows: matrix, BLOSUM62; e-value, 100. To search for transcripts encoding Atg proteins in other crustaceans, we used protein queries of known Atg proteins to search within publicly accessible databases [non-redundant (Nr), expressed sequence tags (ESTs), transcriptome shotgun assembly, and *Daphnia* genome [65] databases] by using NCBI tBLASTn program (http://blast.ncbi.nlm.nih.gov/Blast.cgi), which was restricted to crustaceans (taxid: 6657) and the parameter was set as follows: expect threshold = 10; matrix, BLOSUM62; gap costs, existence = 11 and extension = 1. Nucleotide sequences obtained from BLAST searches were translated into amino acid sequences using the CLC Main Workbench Version 6.0 and then primary sequence analyzed manually based on homology to other known proteins. Protein alignment of crustacean Atg proteins with other previously known Atg proteins was performed using MEGA6 [66] and GeneDoc [67] softwares. Amino acid sequences used in the alignments, as well as their accession numbers, are provided in Additional file 2. All deduced *M. rosenbergii* Atg proteins were finally used for BLASTP search against the Nr protein database for further sequence confirmation. The BLAST hit with lowest e-value for each *M. rosenbergii* Atg protein is detailed in Additional file 1.

### Prediction of protein tertiary structure and critical sites

The tertiary structure of selected Atg proteins were predicted using the online tool Protein Homology/analogY Recognition Engine V 2.0 [68]. Visualization of predicted protein tertiary structure as a 3 dimensional model, illustration of hydrophobicity, superimposition of proteins, and structure labeling were performed using the UCSF Chimera version 1.8.1 [69]. The specific domains and critical sites were predicted by InterPro [70].

### Primary antibodies used in western blotting and immunohistochemistry

Rabbit polyclonal antibody against BECN1 (Sigma-Aldrich, USA) was produced using synthetic peptide corresponding to amino acid positions 329–345 of human BECN1 as immunogen. Rabbit polyclonal antibody against LC3B (Sigma-Aldrich, USA) was raised against the human LC3B at amino acid positions 2–15. Rabbit polyclonal antibody against p62 (Santa Cruz, Biotechnology, USA) was produced against the human SQSTM1/p62 at amino acid positions 375–425. Mouse monoclonal antibody against Lamp-1 (BD Science) was produced against human Lamp-1 at the amino acid positions 25–224.

### Western blot analysis of Atg8/LC3, Atg6/BECN1, and p62/SQSTM1

Fresh tissues were homogenized and sonicated in a lysis buffer (0.2 % NaDoc, 1 mM Na_3_VO_4_ and 50 mM NaF) and then centrifuged at 10,000×*g*, 4 °C, for 1 h to obtain tissue extracts. The supernatants were collected and protein concentrations measured with the Bradford reagent (Sigma). For a positive control, human squamous carcinoma cells (A431 cell line) were sonicated in lysis buffer and protein concentrations measured. Subsequently, 30 μg of total proteins were separated on a 15 % SDS-PAGE gel and transferred onto a polyvinylidene fluoride membrane (GE Healthcare Life Sciences). Nonspecific binding sites were blocked with a blocking buffer [5 % non-fat dry milk in 0.2 % Tween 20 in 0.1 M phosphate-buffered saline (PBS), pH 7.4] for 2 h at room temperature. The membranes were incubated overnight at 4 °C with the following primary antibodies: either rabbit anti-LC3 (dilution 1:500), rabbit anti-p62/SQSTM1 (dilution 1:200), or rabbit anti-BECN1 (dilution 1:500). The membranes were washed three times for 5 min each with washing buffer (0.01 % Tween 20 in 0.1 M PBS) and then incubated for 2 h at room temperature in the peroxidase-conjugated secondary antibody [goat anti-rabbit IgG-HRP (SouthernBiotech) dilution 1:5000]. Antigen-antibody binding was detected using an enhanced chemiluminescent substrate (Thermo Scientific) and exposed to Amersham Hyperfilm ECL (GE Healthcare Life Sciences).

### Immunoperoxidase staining of Atg proteins in the ovary of *M. rosenbergii*

Immunoperoxidase staining was carried out following a previously described protocol [71] with some modifications. Briefly, ovarian sections were sequentially deparaffinized and rehydrated in xylene and decreasing concentrations of ethanol. Endogenous peroxidase was blocked by immersing sections in 3 % H_2_O_2_ in methanol for 30 min. Subsequently, the sections were immersed in 1 % glycine in 0.1 M PBS to block free aldehyde groups and then incubated in a blocking serum (10 % bovine serum albumin in 0.1 M PBS, pH 7.4) for 2 h at 4 °C to block non-specific bindings. Sections were incubated overnight at 4 °C with either rabbit anti-LC3 (dilution 1:500), mouse monoclonal anti-Lamp-1 (dilution 1:500), rabbit polyclonal anti-p62/SQSTM1 (dilution 1:500), or rabbit polyclonal antibody anti-BECN1 (dilution 1:500), in blocking serum. After washing with PBS, sections were incubated in secondary antibody, either goat anti-rabbit IgG-HRP (Abcam) or goat anti-mouse IgG-HRP (SouthenBiotech) at the dilution 1:500 in blocking serum, for 2 h at room temperature. Negative controls were performed by omitting the primary antibodies. The color was developed with an AEC substrate kit (Invitrogen). Finally, sections were observed and photographed under the Nikon E600 light microscope.

## Abbreviations

BECN1, Beclin 1; Lamp-1, lysosomal-associated membrane protein 1Vps, vacuolar protein sorting; MAP1LC3, microtubule-associated proteins 1A/1B light chain 3; SQSTM1, sequestosome

## Additional files

Additional file 1: List of predicted Atg proteins identified in *M. rosenbergii* transcriptomes. The unigene identification number (ID), deduced amino acid sequence, and BLAST hit protein with lowest e-value of each predicted MroAtg are given. (XLSX 45 kb) Additional file 2: List of Atg proteins used in the amino acid sequence alignment. (DOCX 34 kb)
