# Supplementary material for: Transcriptomic analysis of the autophagy machinery in crustaceans
Source: BMC Genomics. 2016 Aug 9;17:587. doi: 10.1186/s12864-016-2996-4 (PMC4979118; doi:10.1186/s12864-016-2996-4)
Supplement: Additional file 2: — List of Atg proteins used in the amino acid sequence alignment. (DOCX 34 kb) [file 12864_2016_2996_MOESM2_ESM.docx]

**Additional file 2: List of Atg proteins used in the amino acid sequence alignment**

(Sequences are present in fasta format. The sequence name is provided as ‘species name_protein name_GenBank accession number’.)

**1. Atg6/BECN1**

>*Macrobrachium rosenbergii­_*Atg6

MDPRVTVNFVCQRCTQPIHLDAGFSHLNEHTLAELALPIYQGIDADFDPSDESLDKLVPPLRLSDSGHHNHGFMLVGESYDTEKTQPLKVVTGLYDLVSSNSDIDHPLCEECTDALLDMMDTTLSRTQKQAQMSQQLLASLNKMPDEDVTALENELKSLELEEERLLVELAETQKEQEVVMTILEKHEEEKARLQDEEERYWREYCSHKYQLTQMEDENRSLKNQLQYTQNQLERLKKTNVFNTTFHIWYSGHFGTINNLRLGRLPTAPVTWPEINAAWGQTVLLLASLARKIGLKFSKYRLVPFGNHSYIEVIGENRELPLYGSGGFRFLWDTKFDAGMVAFLECVAEFQQHVEAQGGQSAKFCLPYRINKHFIEDPTSNNSMSIKMQFNSEEQWTKALKFLLTNLKWGLTWVSSALFSSSQGEN

>*Pontastacus leptodactylus*_Atg6_GAFY01009424

MDPRVTVNFVCQRCLHPIHLDVGFSHLNEHTLAELALPIYPGEEVEFDPSDLDKLVPPLQLTDSGHHPHGFTLVGESGDTEKTQPLKVATGLYDLVSSNSDIDHPLCEECTDALLDLMDTTLSRTQKQAQMSQQLQASLADMPNTDVTALENELKNLQLEEERLIKELEEVEIEQDSAQKVLEKHEEEKARLEDEEERYWREYCTHKSQLTAMEDENRSLKSQLSYTQNQLERLKKTNVFNTTFHIWHSGHFGTINNLRLGRLPTAPVTWQEINAAWGQTVLLLASLARKIGLKFSKYRLVPYGNHSYIEVIGENRELPLYGSGGFRFLWDTKFDAGMTAFLECVAEFQQHVEAQGGQAARFCLPYRINKHCIEDPTSGNSLSIKMQFNSEEQWTKALKFLLTNLKWGLTWVSSALFSSTQVET

>*Calanus finmarchicus_*Atg6_GAXK01132343

MSDLQGGINYLCQRCQQPLRLSTTLYSMDEHTLAELSLPISPAQNIDIASQAASLDRLVLPARLCESGGLAASGGMNNGFTLVGESGQASAMSHRLKVTSQLFDMLSDNSSVDHPLCEECTDTLLQSLEQQLELAEQDSQQYQEYLNKLNIETEDSSEVGELEQELSRLQVEEGELVQELEQLKAENATIDSDLEKQTLAREQLEQQEEQYWRQYSQHKQQLLLAEDEYRSLDCQLRHSQSQLDKLKKTNVFNATFHIWHSGHFGTINGFRLGRLPSIPVDWNELNTAWGQTVLLLHSLVKRIKLQLQRYQLVPYGNYSYVKVLADDKDLPLYGSGGFRLIWDTKFDAGMAAFLDCLQQFKEEVERGDSGFCLPYEMEKGKIRDPATGNTFSVKMQFNSEEQWTKAMKYMLTNLKWGLAWVTSKYTKQE

>Daphnia pulex_Atg6_EFX77818

MEEDGRVVFCCQRCWTPLKLDTGFSCSLDEHTIAELSLPAVRTPELDYTSQASSLDNYVPSRQDHLSINQGFTFVGHAIAGNSGNTNIGHLSHYIRKSTRLFDLVSSTSDIDHPLCEDCSDSLLILLEQQLFQSEEECLEYKQFLAKITKETEDENLSNLEVIEKELLCLQAEELELKNELKELVHKHELITKEIEAEIEEENNVKQEEEKYWKSYSVHRNQQFQVLDEQISLECQLRFTRSNLDRLKQTNAFNAAFHLWHMGHFGTINGLRMGRLPSVPVDWAEINAAWGQVTILLSALARKVNLVFQRYKLVPFGSQSSIEDLVENKVFPLYGSGGFRFLWDAKFDSGMCSFLDCLQQFQQKVEGAKQSEDDTGRLNFQFPYRMERGRIEDRATRQWYSIKIQFNSEEQWTKALKFMLTNLKWGVAWVAAQNSLPELL

>Drosophila melanogaster_Atg6_NP651209

MSEAEKQAVSFACQRCLQPIVLDEQLEKISVHAMAELSLPIYGDNGNTLDPQDASSFDHFVPPYRLTDSINGTGFMLVSDGRDNKKMSAAFKLKAELFDCLSSNSEIDHPLCEECADSMLEIMDRELRIAEDEWDVYKAYLDELEQQRVAPNVEALDKELDELKRSEQQLLSELKELKKEEQSLNDAIAEEEQEREELHEQEESYWREYTKHRRELMLTEDDKRSLECQIAYSKQQLDKLRDTNIFNITFHIWHAGHFGTINNFRLGRLPSVSVDWSEINAAWGQTVLLLSALARKIGLTFERYRVVPFGNHSYVEVLGENRELPLYGSGGFKFFWDTKFDAAMVAFLDCLTQFQKEVEKRDTEFLLPYKMEKGKIIDPSTGNSYSIKIQFNSEEQWTKALKFMLTNLKWGLAWVSSQFVSP

>Crassostrea gigas_BECN1_EKC28450

MATIKVESKSGTTHVSFVCQRCRQPLKLDHSFNTLDRQLLAELSAGPFLTGQFSSLLDDVEVDENYSKRDITSTPEPDEDAGDFLLLGETSPGNMDNLSHRIRVSSALFDVMSGQSEIDHPLCEECTDNLLDQLDNQLKITEDECKDYREFLENLDSNHTEEDGSNLDVELQQLQAEEQSLRQQLQNLETEQEHTEALLEKEREISQKLQDEEDKYWKEYNEYKRQVQELEDEQRSVDNQLKYAQTQLDKLKKTNVFNTTFHIWHSGHFGTINNFRLGRLPSVPVDWNEINAAWGQTVLLLNSLAKKMNLTFQRYRLVPFGNHSYIESLSDKSKELPLYGSGGFRFFWDTKFDQAMVAFLDCLQQFKEEVEKGDTGFCLPYKMEKGKIEDSSTGTSYSIKIQFNSEEQWTKALKYMLTNLKWGLAWVSSQFANK

>Strongylocentrotus purpuratus_BECN1_XP786368

MTSFIHSSDSQRSAPLIAGGGSSGKATPLLQSTDSSEPFDVNRDDGVARKVVAPRLSSHDSGQDFTLLGETASSRMESLSHRVKVASQLFDVMSGQSDIDHPLCEECTDSLLDQLDQQLKITEDECKHYRESLEKLTEAEGRGESNEDLEKELCKLEGEEGEMINELEGIEQERKDIQEEMKKQHNELEELKLEEERYWQEYNEYKRQLLEFQEEQRSVDNQLKYTQTQLERLNKTNVFNSTFHIWHNGHFGTINGFRLGRLPSVAVEWSEINAAWGQTVLLLHSLARKMNFTFKRYRLVPYGNHSHLESLTDKSKQLPLHGSGGFRFFWDTKFDQSMVAFLDCLQQLEEEVERGDSSFCLPYKMTNGKLEDTAAGQTYSIKIQFNSEEQWTKALKFMLTNLKWALAWVSSQFTK

>Caenorhabditis elegans_BECN1_NP500844

MTTQRSHICLNCQHPLRLDFTQRRPDSADSEKKSETVITEALTGHSRNLMKLISDAQFPSDAPVCNDCSDALRNEMDAQVATLDDEIKTYQTYINYLKENHPTTSIPDLKAKLQNVSDEEKELEQQLKKLLAEEEQLDLDLQTKRRTAEAASEKSGELWKKYRDNLRQVFEDQDELHSLEAERQYAEVQHRKLTDTNVLDLCFHIWVDGIVGEINGFRLGYLKDAPVEFTEINAALGQIVLLLEILLERIGVQHHELMPVAMGSHSYIKLRRNGIDMETYALYGQGTPLSGSSGIDPGIRRFLQLLEFLLKELKDRNKNFKPPYQIHADSLVDNGVKYNAVMTLNTDVRWTRAMALMLTDLKAACAQCDALRSPI

>Danio rerio_BECN1_NP957166

METLRFSSNTMQVSFVCQRCNQPLKLDTSFNVLDRMTIHELTAPLVMVTANKQQDSGESSSFPEETFLENKQDGVARKFIPPARMMSAESTNSFTLIGEASDGGTMENLSRRLKVTSNLFDIMSGQTDIDHPLCEECTDTLLDHLDTQLNITENECQNYKSCLELLSQLPEEEEASLLNALQQLKQEEESLIQELESIETKREAVAKELDEGRNHSQLMDTEELRYQKEYCEFKRQQLELDDDLKSVDNQMRYCQIQLDKLKKTNVFNATFHIWHSGQFGTINNFRLGRLPSVPVEWNEINAAWGQTVLLLHALASKMGLCFQRYQLVPYGNHSYLESLSDKSKELPLYCSGGLRFFWDNKFDHAMVAFLDCVQQFKEEVEKDDTGFCLPYRMDVDKGKIEDTGGSGGSYSIKTQFNSEEQWTKALKFMLTNLKWGLAWVSSQFYNR

>Xenopus tropicalis_BECN1_NP001029112

METSKSSTMQVSFVCQRCSQPLKLDTSFKILDKVTMQELTAPLVTTAAVKPGDIQEVDSNIEETFAENRTDGVSRRLIPPARMMSTESATSFTLIGEASDGGTMENLSRRLKVTGDLFDIMSGQTDVDHPLCEECTDTLLDQLDTQLNITENECQNYKRCLEILERMNEDDKEKLEAKLKELAEDEDRLIQELEEVERNRELVAKDIEKVREEAERLEQEEARYQKEYSEFKRQQLELDDDLKSVENQMRYAQIQLDKLKKTNVFNATFHIWHSGQFGTINNFRLGRLPSVPVEWNEINAAWGQTVLLLHALANKMGLQFQRYRLMPFGNHSYLESLTDKSKELPLYCSGGLRFFWDNKFDHAMVAFLDCVQQFKEEVEKGDTGFCLPYRMDVEKGKIEDTGGSGGSYSIKTQFNSEEQWTKALKFMLTNLKWGLAWVSSQFYNK

>Mus musculus_BECN1_NP062530

MEGSKASSSTMQVSFVCQRCSQPLKLDTSFKILDRVTIQELTAPLLTTAQAKPGETQEEEANSGEEPFIETRQDGVSRRFIPPARMMSTESANSFTLIGEASDGGTMENLSRRLKVTGDLFDIMSGQTDVDHPLCEECTDTLLDQLDTQLNVTENECQNYKRCLEILEQMNEDDSEQLQRELKELALEEERLIQELEDVEKNRKVVAENLEKVQAEAERLDQEEAQYQREYSEFKRQQLELDDELKSVENQVRYAQIQLDKLKKTNVFNATFHIWHSGQFGTINNFRLGRLPSVPVEWNEINAAWGQTVLLLHALANKMGLKFQRYRLVPYGNHSYLESLTDKSKELPLYCSGGLRFFWDNKFDHAMVAFLDCVQQFKEEVEKGETRFCLPYRMDVEKGKIEDTGGSGGSYSIKTQFNSEEQWTKALKFMLTNLKWGLAWVSSQFYNK

*>Homo sapiens*_BECN1_NP003757

MEGSKTSNNSTMQVSFVCQRCSQPLKLDTSFKILDRVTIQELTAPLLTTAQAKPGETQEEETNSGEEPFIETPRQDGVSRRFIPPARMMSTESANSFTLIGEASDGGTMENLSRRLKVTGDLFDIMSGQTDVDHPLCEECTDTLLDQLDTQLNVTENECQNYKRCLEILEQMNEDDSEQLQMELKELALEEERLIQELEDVEKNRKIVAENLEKVQAEAERLDQEEAQYQREYSEFKRQQLELDDELKSVENQMRYAQTQLDKLKKTNVFNATFHIWHSGQFGTINNFRLGRLPSVPVEWNEINAAWGQTVLLLHALANKMGLKFQRYRLVPYGNHSYLESLTDKSKELPLYCSGGLRFFWDNKFDHAMVAFLDCVQQFKEEVEKGETRFCLPYRMDVEKGKIEDTGGSGGSYSIKTQFNSEEQWTKALKFMLTNLKWGLAWVSSQFYNK

**2. Vps34/SQSTM1**

**>***Macrobrachium rosenbergii*_Vps34

MEPDSDKFWYVYSSDLDVDIQIKIGTLDGKREKPNYKSLLEDPMLKYSGLYQPGCSDLYVSCQVYSDGRELTLPVRTSYKSFSARWNWNEWLTLPLRFSDLPRNAVLALTIYDTYGPRRNEPIGGTTISFFGKHGVFRQGLHDLRVWPGQVAHPIHTPGKGQDHGKDQMQRLAKLAKKHRNGHIMKVDWLDRLTFREIELINEQEKRSSNSMYLMVEFPRVEHEGVIYSIVWWESGGSMVYDHRTSGNIVRMPDPEIPLENLQEAKHHKLARSNRSGVTDRDLKPNSAIRDILNTIMAYPPIKQLSNDDQDTVWKYRFYLSSQKKALTKFLKCVNWKLPGETKQAIELLSRWAPPDADDALELLGPGFTHPDVRRYAVERLSLAADEDLLLYLLQLVQALKYEALEGVNMSLCDVSHYSSASDTSADTCGTGGTPSDAPAQEMIDSPEPAGGELTQSISSSSMEESEAATIQRSPTSSFNAEVETSDQFVSEPMCELLPKLAEETDTNLDLASFLIHRATKNPTLANYLYWYLLVECEEHEPGLKHDTKVREMYLWVLRRFKLALQQGCREARASRQSLMRQHHFMDQLVALVKCVAKESSNRMRRIEKLQSLLGDPEAFKTNFANFEPLALPLDPEVIVTGIIPDRASLFKSALEPCRLTFKTVTNEEYVAIFKHGDDLRQDQLIIQMITLMDKVLRRENLDLKLTPYKVLATSSKHGFVQFVESTAVAEVLRAEGSILQFFRKHSPSENGPCGIAPEVMDTYIKSCAGYCVITYLLGIGDRHLDNLLLTKRGNLFHIDFGYILGRDPKPLPPPMKLSKEMVEGMGGAQSEHYHEFRKLCYTSFHQLRRHANLILNLFSLMVDANVPDIALEPDKTVKKVEDKFRLDLSDEEAVSYMQGLIDDSVNAVVAAMVEQLHKIAQYIRK

>*Pontastacus leptodactylus*_Vps34_GAFS01011900

MEPDSDKFWYVHSCDLDVDIQVKIGTLDGKREKPNYEALLKNPLLKYSGLYQSGCSDLYVSCQVFSDGRELTLPVRTSYKPFSARWNWNEWLTLPLRFSDLPRNSVLALTIYDTYGPRINEPIGGTTISFFGKHGVFRQGLHDLRVWPNRIADHVLTPGKGQDHGKDQMQRLAKLAKKHRNGQMMKVDWLDRLTFREIELINEQEKRSSNSMYLIVEFPRVEHEGVIYSIVWWERGGNVEYEHRTCGNMVRMPDPEIALENLQEAKHHRLARSLRSGVTDKDLKPNSAIRDSLHTIMEYPSTKQLTSDEQDMVWKYRFYLSTQKKALTKFLKCVNWKLLGEAKQAIELLGRWVPPDVDDALELLGPGFTHPDVRRYAVERLSIASDEDLLLYLLQLVQALKYEALDNVDMSLCDVSHYSSASDTSGDTCHTEERTPEDSMQDRLCESTGSTGGDLTQSASSSLEESEAATIQRSPSSSLLAEPDSSDPSATAPDSGLLPELAEMDSELDLASFLIHRATRNPTLANYLYWYLLVECEDQEAGLKHDTKVREMYLWVLRRFKLALQQGSREARSSRLLLTRQHHFMDQLVTLVKAVGRESGNRMRKIEKLQTLLADSENLKINFANFEPLALPLDPEIIVTGIMPDRATLFKSALEPSRLTFNTVTGEEYVAIFKHGDDLRQDQLIIQIITLMDRLLRRENLDLRLTPYKVLATSSKHGFVQFIESHAVADVLRTEGGIQNFFRKHAPSETGPYGIAPEVMDTYIKSCAGYCVITYLLGIGDRHLDNLLLTRNGNLFHIDFGYILGRDPKPLPPPMKLSKEMVEGMGGAQSEHYHEFRKLCYTAFHQLRRHANLILNLFSLMVDANVPDIALEPDKTVKKVEDKFRLDLSDEEAVSYMQGLIDDSVNAVVAVVVEQLHKFAQYIRK

>*Penaeus monodon*_Vps34_JR226010

DAEIQVKIGNLDGKRDNPNYEALLKDSLLKYSGLYQAGCSDLYVTCQVICDGRQLTLPVRTSYKSFSARWSWNEWLTLPLKFRDLPRNALLALTVYDSLGPRRIERVGGTTISFFGKQGVFRQGLHDLRLWPGRVGDETTPGKGHDRGKDQMVRLAKLAKKHRNGQMMKVDWLDRLTFREIEVINEHEKRSSNAMYLMVEFPRVEHEGVIYSVVWWERGGEVEYRHRSAGSVVRMPDPEIGLENLQEAKHHRLARSLRSGVTDRDLKPNLAIRDALHTIVDYPPTKQLTSDEQDTVWKYRFYLSSQKRLSSQKRALTKFLKCVNWKLTGEAKQAIELLNRWAPPDADDALELLGPGFTHPEVRRYAVKRLSHASDEDLLLYLLQLVQALKYEAIEGVDTSLCDVSHYDENEEQEEEDDEEEEEGMMKGEEVREEKKIGKRPQEQGGRGIIGEDMYKDEEEDESEESESDTATLKQSGSSSLLADVEGGGVEKVEDKGSEKGEKGITATASNDSDRDPKTDSMETQKVSPTTPNQTEDSSQLRDPNLIASMEADPKGLDLASFLIQRATKNPTLANYLYWYLIVECERDEPGLKHEAKVVEMYEWVVRRFRAALWAGGRAARATRRDLARQTHFLEQLVALVKVVAKESNRVRRIEKLQSLLADSAAYKVNFSAFNPLRLPLDPEVIVTGIVPEKATLFKSALEPSRLTFVDVEGKEYVAIFKHGDDLRQDQLIIQMLTLMDRLLRRENLDLRLTPYKVLATSSRHGFVQFVESLSVAEVLRTEGSILNFFRRHAPSETGPQGISSEVMECYIKSCAGYCVMTYLLGVGDRHLDNLLLTKDGNLFHIDFGYILGRDPKPLPPPMKLSKEMVEGMGGQQAESYHEFRRLCYTAFHQLRRHANLILNLFSLMVDANVPDIALENDKAVKKVEDKFRLDLSDEEGASFVQGLIDDSVNAVVAVVVEQLHKFAQYIRK

>Daphnia pulex_Vps34_EFX81394

MAETGTSGTTRFHYLYSCDLDINIQIKIGSLEGRLERPTYEDLLKDPLLKHSGIYQDTCPELCIKTQIWADNEPNTLPITTSYKSFTTRWNWNEWINLPMKYCDLPRNSQLCLTIYECCGPGKYVPVGGTTLPLFGKLGVYKQGLHDLRIWRNQVADGNLPSKTPAKVKDSGNEVMESLSKLTKRHHTGRIPQSDWLDRLTFREIELINEREKRTSNYMYLMVEFPRLEYHGVEYTVVYYEKGGDDMITQLTQSEYAVLNDDEILLENVIEGKHHKLARSIRSGLTDRDLKPDAGTRDQLHAIVDSPPTKTLASEEQDLVWRFRFYLSNQKKALTKFLKCVKWTSMSEARQAVDLMNRWAPIDPEDALELLGPNFTYSTVRKYAVSRLSHAPDEDLQLYLLQLVQALKYEDFDEICAGLDPRSLMLSVRSFNSADSFPSPEKAGHDVSIDERFNSEMESPVLESSPLLSAETPTPVRFINSESGEYSVDLPTFLIQRACANSALVNYFYWYLMIECEDPEPNVKHDARVREMYLVVMKKFLQALAKGGKEWRRRRAILNRQQQFLDRLVSLMKAVARESGNRKKKMERLRTLLADRDFFKGGTFLEFEPLPFLLDPELLIKGIVAEKATLFKSQLMPACLTFSTVSGQEYVAIFKHGDDLRQDQLILQMISLMDRLLRRENLDLKLTPYRVLATSSRHGFVQYIDSVSVREVSDTEGSILNFFRKYHPSESGPYGIAAEVMDCYIRSCAGYCVITYLLGVGDRHLDNLMLTKSGKLFHIDFGYILGRDPKPLPPPMKLSKEMVEAMGGVHSEHYTQFRKLCYTTFLHLRRHANLILNLFALMVDASVPDIALEPDKTVRKVQDKFRLDLNDEEALRYMQNLIDVSAAAVMAALVEQLHKIAQYWRK

>Caenorhabditis elegans_Vps34_NP001020954

MRATPTESFSFVYSCDLQTNVQVKVAEFEGIFRDVLNPVRRLNQLFAEITVYCNNQQIGYPVCTSFHTPPDSSQLARQKLIQKWNEWLTLPIRYSDLSRDAFLHITIWEHEDDEIVNNSTFSRRLVAQSKLSMFSKRGILKSGVIDVQMNVSTTPDPFVKQPETWKYSDAWGDEIDLLFKQVTRQSRGLVEDVPWLDPFASRRIEMIRAKYKYSSPDRHVFLVLEMAAIRLGPTFYKVVYYEDETKNMRVSTSVNGGVGIVSACTRYCVADPELLLESLAEVKHSAMTRRIRDVEDERHRQVKPNKQAKDRLETIVNLPSSQVLTREQRDLVWKFRHYLRQFPKALNKYLRSVNWVHPQEVKTALALMNDWELIEAEDALELLSSAFTHPAVRAYSVSRLLEAASPEQVLLYLPQLVQALKYEQGQQLPEEGNPVPVVSEEEGKIPSVATTPTEELEGRDMTVVTKKEARKAASGDLATFLIDYALASPKVSNYLYWHLKTEIESTKESKEEHSKMYQNIQDRLMEALVKRPDTRAQVDSLHQQQIFVEDLIILMNEAKARGGRLNESKSAEFRTMLSRAKHMLDLKGVHLPLDPSFRLSSVIPDTASFFKSEMMPAKISFKVLQPNGKADRNIPEEYTVIFKTGDDLRQDQLIQQMVRLIDIILKKGQLDLKLTPYLVLSTGVGQGFVQCIKSKPLRAIQEQYKAHKMDCIREAMKELRPGDGPFGIEPNVIDNYVRSLAGYSVIMYILGLGDRHLDNLLLCENGKLFHVDFGFILGRDPKPMPPPMKLTSEMVQVMGGVKSKQFLEFVQHVDSAYRILRRHSNVLLNLFSLMLDAGIPDIAAEPDKAIFKIEQRLRLDLSDEAATKHIFTQIESSLNAKMAMISDIIHAYKQNLM

>Danio rerio_Vps34_NP001017550

MDTDKFNYVYSCDLDINVQLKIGSLEGKREQKSYKALLEDPMLRFSGLYQESCSDLYVTCQAFAEGKPLALPVRTSYKAFSTRWNWNEWLRLPVKYPDLPQSAQVTLTVWDVYGPGRATPVGGTTVTLFGKYGMFRQGMHDLKVWPGVEGDGTEPTSTPGRTSSSLAEDQMGRLAKLTKAHRQGHMVKVDWLDRLTFREIEMINESEKRSSNFMYLMVEFPRVKSGEREYSIVYYEKDADESSPLPTSSDIVKVPDPQMCMENLVESKHHRLARSLRSGPSDHDLKPNAATRDQLNIIVSYPPTKQLSSEEQDLVWKFRYYLTTQEKALTKFLKCVNWDLPQEAKQALELLGKWRPMDVEDSLELLSSQFTNPTVRRYAVARLQQADDEDLLMYLLQLVQALKYENFNDIQGGLEPANKRDNQGGLTESSTTGDLDSSQLASAIAVMPSSQKGKEGTDGENLEQDLCTFLISRACKNSTLANYLYWYVIVECEDQDTQQRDPKTHDMYLNVMRRFSQALLKGDKSVRVMRSLLASQQTFVDRLVQLMKAVQRESGNRKKKTERSQSLLADNEKVNLSEIEPIPLPLEPQIRIKGIIPETATLFKSALMPAKLIFKTEDGEQYPVIFKHGDDLRQDQLILQIISLMDKLLRKENLDLKLTPYKVLATSTKHGFMQFVQSVPVAEVLATEGNIQSFFRKYAPNDKGPYGISSEVMDTYVKSCDTPISF

>Xenopus tropicalis_Vps34_XP002935097

MGESDRFYYVXXXXLNDSLYLHRGSLEGKREQKSYKAVLEDPMLKFSGLYQETCSDLYVTCQVFAEGKPLALPVRTSYKAFSTRWNWNEWLKLPVKYADLPRSAQVALTIWDVYGPGKAIPVGGTTVSLFGKYGMFRQGMHDLKVWPNIEADGSEPTKTPGRTSSSASEDQMSRLAKLTKAHRQGHMVKVDWLDRLTFREIEMINESEKRSSNFMYLMVEFPCVKSDEKEYGIVYYEKDGDESTPISSSSEIVRVPDPQMSMENLVESKHHKLARSLRSGPSDHDLKPNAATRDQLNIIVSYPPTKQLTSEEQDLVWKFRHYLTNQEKALTKFLKCVNWDLPQEAKQALELLGKWKPMDVEDSLELLSSHFTNPTVRRYAVARLQQADDEDLLMYLLQLVQALKYENFEDIKSGLEPTKKDSQGPMLESMTTSGINPESDSSQVISNPLPSAVSSPAPSSKTKDGLDAENLEQDLCTFLISRACKNSTLANYLYWYVIVECEDQDTQLRDPKTHEMYLNVMRRFSQALLKGDKSVRVMRSLLATQQTFVDRLVHLMKAVQRESGNRKKKNERLQALLGDNEKMNLSEFEPIPLPLEPQVKIRGIIPEKATLFKSALMPAKLYFKTEDGGRYPVIFKNGDDLRQDQLILQIISLMDKLLRKENLDLKLTPYKVLATSTKHGFMQFIQSVPVAEVLATEGSIQNFFRKYAPSEKGPYGISAEVMDTYVKSCAGYCVITYILGVGDRHLDNLLLTKTGKLFHIDFGYILGRDPKPLPPPMKLNKEMVEGMGGTQSEQYQAFRKQCYTAFLHLRRYSNLILNLFSLMVDANIPDIALEPDKTVKKVQDKFRLDLSDEEAVHYMQTLIDDSVNALFAAVVEQIHKFAQYWRR

>Rattus norvegicus_Vps34_NP075247

MGEAEKFHYIYSCDLDINVQLKIGSLEGKREQKSYKAVLEDPMLKFSGLYQETCSDLYVTCQVFAEGKPLALPVRTSYKPFSTRWNWNEWLKLPVKYPDLPRNAQVALTIWDVYGPGRAVPVGGTTVSLFGKYGMFRQGMHDLKVWPNVEADGSEPTRTPGRTSSTLSEDQMSRLAKLTKAHRQGHMVKVDWLDRLTFREIEMINESEKRSSNFMYLMVEFRCVKCDDKEYGIVYYEKDGDESSPILTSFELVKVPDPQMSMENLVESKHHKLARSLRSGPSDHDLKPNATTRDQLNIIVSYPPTKQLTYEEQDLVWKFRYYLTNQEKALTKFLKCVNWDLPQEAKQALELLGKWKPMDVEDSLELLSSHYTNPTVRRYAVARLRQADDEDLLMYLLQLVQALKYENFDDIKNGLEPTKKDSQASVSESLSSSGVSSADIDSSQIITNPLPPVASPPPASKSKEVSDGENLEQDLCTFLISRACKNSTLANYLYWYVIVECEDQDTQQRDPKTHEMYLNVMRRFSQALLKGDKSVRVMRSLLAAQQTFVDRLVHLMKAVQRESGNRKKKNERLQALLGDNEKMNLSDVELIPLPLEPQVKIRGIIPETATLFKSALMPAQLFFKTEDGGKYPVIFKHGDDLRQDQLILQIISLMDKLLRKENLDLKLTPYKVLATSTKHGFMQFIQSVPVAEVLDTEGSIQNFFRKYAPSETGPNGISAEVMDTYVKSCAGYCVITYILGVGDRHLDNLLLTKTGKLFHIDFGYILGRDPKPLPPPMKLNKEMVEGMGGTQSEQYQEFRKQCYTAFLHLRRYSNLILNLFSLMVDANIPDIALEPDKTVKKVQDKFRLDLSDEEAVHYMQSLIDESVHALFAAVVEQIHKFAQYWRK

>*Homo sapiens*_Vps34_NP002638

MGEAEKFHYIYSCDLDINVQLKIGSLEGKREQKSYKAVLEDPMLKFSGLYQETCSDLYVTCQVFAEGKPLALPVRTSYKAFSTRWNWNEWLKLPVKYPDLPRNAQVALTIWDVYGPGKAVPVGGTTVSLFGKYGMFRQGMHDLKVWPNVEADGSEPTKTPGRTSSTLSEDQMSRLAKLTKAHRQGHMVKVDWLDRLTFREIEMINESEKRSSNFMYLMVEFRCVKCDDKEYGIVYYEKDGDESSPILTSFELVKVPDPQMSMENLVESKHHKLARSLRSGPSDHDLKPNAATRDQLNIIVSYPPTKQLTYEEQDLVWKFRYYLTNQEKALTKFLKCVNWDLPQEAKQALELLGKWKPMDVEDSLELLSSHYTNPTVRRYAVARLRQADDEDLLMYLLQLVQALKYENFDDIKNGLEPTKKDSQSSVSENVSNSGINSAEIDSSQIITSPLPSVSSPPPASKTKEVPDGENLEQDLCTFLISRACKNSTLANYLYWYVIVECEDQDTQQRDPKTHEMYLNVMRRFSQALLKGDKSVRVMRSLLAAQQTFVDRLVHLMKAVQRESGNRKKKNERLQALLGDNEKMNLSDVELIPLPLEPQVKIRGIIPETATLFKSALMPAQLFFKTEDGGKYPVIFKHGDDLRQDQLILQIISLMDKLLRKENLDLKLTPYKVLATSTKHGFMQFIQSVPVAEVLDTEGSIQNFFRKYAPSENGPNGISAEVMDTYVKSCAGYCVITYILGVGDRHLDNLLLTKTGKLFHIDFGYILGRDPKPLPPPMKLNKEMVEGMGGTQSEQYQEFRKQCYTAFLHLRRYSNLILNLFSLMVDANIPDIALEPDKTVKKVQDKFRLDLSDEEAVHYMQSLIDESVHALFAAVVEQIHKFAQYWRK

>Saccharomyces cerevisiae_Vps34_NP013341

MSLNNITFCVSQDLDVPLKVKIKSLEGHKPLLKPSQKILNPELMLIGSNVFPSSDLIVSLQVFDKERNRNLTLPIYTPYIPFRNSRTWDYWLTLPIRIKQLTFSSHLRIILWEYNGSKQIPFFNLETSIFNLKDCTLKRGFESLKFRYDVIDHCEVVTDNKDQENLNKYFQGEFTRLPWLDEITISKLRKQRENRTWPQGTFVLNLEFPMLELPVVFIEREIMNTQMNIPTLKNNPGLSTDLREPNRNDPQIKISLGDKYHSTLKFYDPDQPNNDPIEEKYRRLERASKNANLDKQVKPDIKKRDYLNKIINYPPGTKLTAHEKGSIWKYRYYLMNNKKALTKLLQSTNLREESERVEVLELMDSWAEIDIDDALELLGSTFKNLSVRSYAVNRLKKASDKELELYLLQLVEAVCFENLSTFSDKSNSEFTIVDAVSSQKLSGDSMLLSTSHANQKLLKSISSESETSGTESLPIVISPLAEFLIRRALVNPRLGSFFYWYLKSESEDKPYLDQILSSFWSRLDKKSRNILNDQVRLINVLRECCETIKRLKDTTAKKMELLVHLLETKVRPLVKVRPIALPLDPDVLICDVCPETSKVFKSSLSPLKITFKTTLNQPYHLMFKVGDDLRQDQLVVQIISLMNELLKNENVDLKLTPYKILATGPQEGAIEFIPNDTLASILSKYHGILGYLKLHYPDENATLGVQGWVLDNFVKSCAGYCVITYILGVGDRHLDNLLVTPDGHFFHADFGYILGQDPKPFPPLMKLPPQIIEAFGGAESSNYDKFRSYCFVAYSILRRNAGLILNLFELMKTSNIPDIRIDPNGAILRVRERFNLNMSEEDATVHFQNLINDSVNALLPIVIDHLHNLAQYWRT

**3. Atg8/LC3**

>*Macrobrachium rosenbergii*_Atg8

MNSHTKPFRERRNFAQRQRDVEQIREQHPNKVPVIIERYPGEKHLPLLDKTKFLVPDHVTMGELVKIIRRRLQLHPTQAFFLLINQRALASVSNTLAQVYQQHHHEDGFLYMVYASQEVFGQ

>*Macrobrachium* nipponense_Atg8_SRR496753.6860

MNTHAKPFRERRNFAQRQRDVEQIREQHPNKVPVIIERYPGEKHLPLLDKTKFLVPDHVTMGELVKIIRRRLQLHPTQAFFLLINQRALASVSNTLAQVYQQHHHEDGFLYMVYASQEVFGQ

>Litopenaeus vannamei_Atg8_JP427798

MNNQVKPFRERRSFAQRQRDVEQIREQHPNKVPVIIERYPGERHLPLLDKTKFLVPDHVTMGELVKILRRRLQLHPTQAFFLLINQRALASVSNTLAQVYEHHHHEDGFLYMVYASQEVFGQ

>Scylla olivacea_Atg8_GDRN01056303

MKWQYREEHPFEKRRAEGEKIRKKYPDRVPVIVEKAPKARIGDLDKKKYLVPSDLTVGQFYFLIRKRIHLRPEDALFFFVNNVIPPTSATMGSLYQEHHEDDFFLYIAYSDESVYGC

>Eriocheir sinensis_Atg8_FG359980

MNAQVKPFRERRSFAQRQRDVEQIREQHPNKVPVIIERYPGERHLPLLDKTKFLVPDHVTMGELVKIIRRRLQLHPTQAFFLLINQRSLANVSSTLAQVYEHHHHEDGFLYMVYASQEVFG

>Daphnia pulex_Atg8_EFX80203

MSKMTQTSFKERRNYGQRVKDADMVRELHPNKIPIIVERFAGEKNLPLLDKTKFLVPDHVTVAELIKILRRRMQLNPNQAFFLLVNQRSMASISMTMGELFQREQDEDGFLYMVYASQESFGN

>Drosophila melanogaster_Atg8_ NP727447

MKFQYKEEHAFEKRRAEGDKIRRKYPDRVPVIVEKAPKARIGDLDKKKYLVPSDLTVGQFYFLIRKRIHLRPEDALFFFVNNVIPPTSATMGSLYQEHHEEDYFLYIAYSDENVYGMAKIN

>Caenorhabditis elegans_LGG-1(Atg8 ortholog)_ NP495277

MKWAYKEENNFEKRRAEGDKIRRKYPDRIPVIVEKAPKSKLHDLDKKKYLVPSDLTVGQFYFLIRKRIQLRPEDALFFFVNNVIPQTMTTMGQLYQDHHEEDLFLYIAYSDESVYGGEVEKKE

>Danio rerio_Atg8_NP955898

MPSEKTFKQRRTFEQRVEDVRLIREQHPNKIPVIIERYKGEKQLPILDKTKFLVPDHVNMSELIKIIRRRLQLNSNQAFFLLVNGHSMVSVSTAISEVYERERDEDGFLYMVYASQETFGFQ

>Xenopus tropicalis_Atg8_NP989346

MPSEKTFKQRRSLEQRVEDVRLIREQHPTKIPVIIERYKGEKQLPVLDKTKFLVPDHVNMSELIKIIRRRLQLNSNQAFFLLVNGHSMVSVSTPISEVYEREKDEDGFLYMVYASQETFGVKYA

>Mus musculus_Atg8_AAI10309

MPSEKTFKQRRSFEQRVEDVRLIREQHPTKIPVIIERYKGEKQLPVLDKTKFLVPDHVNMSELIKIIRRRLQLNANQAFFLLVNGHSMVSVSTPISEVYESERDEDGFLYMFYASQETFGTAMAV

>*Homo sapiens*_Atg8_NP073729

MPSEKTFKQRRTFEQRVEDVRLIREQHPTKIPVIIERYKGEKQLPVLDKTKFLVPDHVNMSELIKIIRRRLQLNANQAFFLLVNGHSMVSVSTPISEVYESEKDEDGFLYMVYASQETFGMKLSV

**4. p62/SQSTM1**

**>***Macrobrachium rosenbergii*­­_p62

MSEEQSMSVKAFLDAGKDRQEVRRFGLPETLATNFAALREKICTVFNLSPQEIIISWKDCEGDSIVISSDEELVEAIADSLSKQKMQVFRVNVTLRQDDSEQGNQPGQPQGNQQGDIHEGVVCDVCDGPVVGFRYNCVTCQDFDLCGACETKGLHREHKMIRMPKPLSRGEPRFWWKEQSGPGGQYTSHFRMHSGAGGGDSNAYSFSSSTSGPGADAGGWCGWGPWMGRGGSRSCNRNGGRGWRGWWGKNWGGARADAGCWQGQGQTLGGFSSQQQQQQGQQQQQQGQQQQQQHQEQQQQQDQSKEKEQQRSANDNEKECPWTSANGGPCPFAEGHPIPSPERVAEEVQFAAQEAQKMAHHVAHHFASQMATDHVANVMRGIWTAWSGQQPPGVGPAVGQTSHSSSSSSSSASSAGGNNQEDGKSKNSSNGPSQSSHEQFLRNIGESVASMLEPMGIDVDVSVEHNGIRQRCSLSEDDLQARQSPTSKTPDVPKTPDATEPPTVSRDPQPTASSSSNMEVQTDVPEQNPVEMEVSSPPDGEAQMESDVEEWTMVNRDSQSPDKQAPAKPKTPPTQQRVEYPDLRQVETHPNPAVQQALEQMQSMGYSNEGGWLTNLLEMKQGDINQVLDLLQPTHK

>*Pontastacus leptodactylus*_p62_GAFY01003445

MSEERSLSVKVYLDVGGGRQEVRRFALPENLATNFRCLKEKVASVFTLGNKEFAISWKDAEGDLIVISSDDELMEAIADTLSKQNLQLLRINVTVGSRPDVGPNNSVPGNQQGTLHEGVVCDVCEGPVRGFRYRCVSCEDFDLCGACESKGLHAEHKMLRLAKPHVKGSPWFFWRESEDSPGNFTSHFGLSGGTGPTCFTSSSTAGAGTGAGAWSNWGGRGGRHGHRRGCGSRGPGGWAGWWGKNWGGKAGGGCEQSQSMGTGFGQQHQHHRHHQQHHQQQQHQQQQQQQQQQQHQSQQQGNVNSNMHSEFPWTHTCPFMTGECSPQHMAEDAQKMAEDAQKMAEEAAQTAHEFASQMASEHVANVMRGIWTAWTGHQGPGLIKTASTSSSSSSSSSSNGDTQQPKSSKPKGEEDQQQKDTNKEANQTAGEEYLRNVGETVAAMLDPIGIDVEVSVEHNGIRQRCSLSKDDVESRKSPTASFSPSPPKEPESSEQSLASETSANMEVQNDASRQRGGGVDGVGEIMEVCSPQAGPSHNSGEQMEAEQSSSDIEDWTLVNKDANSLSQGAAKSKTPPTERRVAYPDLSVIEAHPNPIIQRALEQMQSMGYNNESGWLTNLLELKQGDINQVLDLLQPVHK

>Daphnia pulex_p62_EFX75305

MANSLPFKCYLLTNNGQDKEIRRFALDSDVVGNFTYLQEKVRVVYPQLLRESFTISYIDEEGDKVTVSSDDELVAALMFAKRKDDEPFRLIVQTTAATSGAKPETATPSAGTGNCQGEIHWGVTCDGCQGAVKGFRYKCFQCPDYDLCGKCESAGQHPGHILIRVTGAMPAAFQAMKHALNGGPEVPHWRRGKHGRYHHQHGHGWNAWCPSGMNVEVDPQTTAAGSNKGASKTEAKPQCPYKFYMDQAKETATTIHAQAKETASIFNEQHPEYLAGLGSTIASVMEGLGFGSVGGSCPRASTQKPEAKKQETKKEETKAEEKDSTAEAKKEEVIIPVVIEKEETPTNAAVVNPYAHLVDAARAVEVASAKAMADFARQAAVQTGVAPKPAETMSFFQTAEAKKDTPFAVACRARMTAPQAPPQAVAEAIAEVVAQAPSQAIAEAVAEAAALAPTQAVADAIAKVAAEAPSQIAAQAVAQAAEDAYKDSLVSQQTVTEPTSVKPKHNESGDWTIVDHGMEHSSSPPPPSVPASQFVGARPKEPTAEKAPLHPDPFISAALETMLAMGFTNEGGWLAQLLEVKGGDIGKALDVLQSQFQRQQHQ

>Danio rerio_p62_NP998338

MTLSVSSSSNEKSTNVILLLMEFLGFRSLHLTLALLVPVIWVHLQWGLQDHLIWGVLLTTLPLTQWCTVCPDYDLCPTCQSKGLHKEHALLPIFHPMANVFEWLPRGKFWRKMRHCMWAQAQAQNQAQNQPQPGPSGAQQNQDAPENPNENGATASSQANVEYLKNIGEEVAAMLSPLGIDVDIDVEHEGKRTKVTPTPPASSGPSSARSDSGSVGLLSRGSGPGSQATEESVSEGTKKDQCSDEEWTHLSAKEVDPSTGELQSLRLEQDGADLPAPLNTASGTSTREGPTGLREAALYPHLPQDADPRLVESLSQMLSMGFTDEGGWLTRLLHTKNYDIGGALDTIQYSKTPGQQK

>Xenopus tropicalis_p62_NP001007894

MPVTVKAYLLGKDESHREIRRFQLELPAAGKEKASVSSCELLANKVTGVFQGLKGGAFQMFYKDEEGDLVAFSTDEELHMGLSLLNEDVFRIYIKERKECKRDHRGHCGQETPQNVVHPNVTCDGCDGPVVGNRFKCLICPDYDLCSTCEKKGIHKEHNMIMFPTPLVYPRSRWFRKMHHGVPPFPWMQGWAYPPRGYPCQNFQQAQESTPPQNETPAEEAAATSDSQSSQNPNVAFLKNVGESVAAMLSPLGIDVDIDVEHGGKRTKVSTSHPCSVDGDSQPNSSTSFTEIQDIASRSDSAPNLMETEHIAKRMKDIALNSPLPQDNEQGENSSSASGGDDDWTHVSSKEVDPSTGELQSLQLMETGQPCSLDPTRSSALPTHAPTGLREAALYPHLPPEADPRLIESLSQMLSMGFTDEGGWLTRLLEAKQFDIGSALDAMQSIRHIPPS

>Mus musculus_p62_NP035148

MASFTVKAYLLGKEEATREIRRFSFCFSPEPEAEAQAAAGPGPCERLLSRVAVLFPTLRPGGFQAHYRDEDGDLVAFSSDEELTMAMSYVKDDIFRIYIKEKKECRREHRPPCAQEAPRNMVHPNVICDGCNGPVVGTRYKCSVCPDYDLCSVCEGKGLHREHSKLIFPNPFGHLSDSFSHSRWLRKLKHGHFGWPGWEMGPPGNWSPRPPRAGDGRPCPTAESASAPPEDPNVNFLKNVGESVAAALSPLGIEVDIDVEHGGKRSRLTPTTPESSSTGTEDKSNTQPSSCSSEVSKPDGAGEGPAQSLTEQMKKIALESVGQPEEQMESGNCSGGDDDWTHLSSKEVDPSTGELQSLQMPESEGPSSLDPSQEGPTGLKEAALYPHLPPEADPRLIESLSQMLSMGFSDEGGWLTRLLQTKNYDIGAALDTIQYSKHPPPL

>*Homo sapiens*_p62_NP003891

MASLTVKAYLLGKEDAAREIRRFSFCCSPEPEAEAEAAAGPGPCERLLSRVAALFPALRPGGFQAHYRDEDGDLVAFSSDEELTMAMSYVKDDIFRIYIKEKKECRRDHRPPCAQEAPRNMVHPNVICDGCNGPVVGTRYKCSVCPDYDLCSVCEGKGLHRGHTKLAFPSPFGHLSEGFSHSRWLRKVKHGHFGWPGWEMGPPGNWSPRPPRAGEARPGPTAESASGPSEDPSVNFLKNVGESVAAALSPLGIEVDIDVEHGGKRSRLTPVSPESSSTEEKSSSQPSSCCSDPSKPGGNVEGATQSLAEQMRKIALESEGRPEEQMESDNCSGGDDDWTHLSSKEVDPSTGELQSLQMPESEGPSSLDPSQEGPTGLKEAALYPHLPPEADPRLIESLSQMLSMGFSDEGGWLTRLLQTKNYDIGAALDTIQYSKHPPPL

>Apis mellifera_p62_XP392222

MKMFKAYLQNSDFSIKEIRKFNLYPSNLSDKFEVLCNKIKELFPELNHKSFTISWKDNDGDQIVMSSEDELKIAFNEIRNKEVETKYLAIYIKPTIQKEQKSTTNPYQNDLNEKVIHFGITCDGCDNDIIGFRYKCIQCEDYDLCAQCEAAGIHPHHCMIRMPQPLKWHHSRSLHHHLRKIFKKNGVHLNKKTSSNENKESQGIHCNIYPWFETYAPYLNNFIDALLEVHNVESNSSKVEKKEESKNNSHIDDNDSKKFPGEGRKLFDDTKDDKESVSDVASTTSQDSNPPKVTADEWTIIDTKDTTEANHTASTSSNMNETNEKEKSSSTAPSAPNGTSIYPELPKEKIIHHQNPIINEAVENMIRMGFSNQGGLLTYLLDAENGDINKVLEILQPTNKR

>Strongylocentrotus purpuratus_p62_XP011664187

MSMTVKAYLKRGENANAEIRRFVIDVAVSSNYEYLSKKVAQVFPSLGDPDYFSLSWKDSEGDNITFSSDDELVEALGQINDDTFRIYVKEKKRCRREIRDAPSPGTSTDDGAGQEEQAYHPGVICDGCESRIRGPRFKCITCPDYDLCKQCESRGIHPDHSFVKFRKPQVGRSHHGGFFNRPGMFRPFGHPGWRHWWRHQQQQQQQGAGDASTTQNEQTQGTGPQINVGCTMGPGLFGGPPPPPHGPHMPPPPPHGPNMPPPPHEFLRDIGQTVAQMLDPLGIDVDIDIDNQGNRTHCAGEPNFCGGGGGGHGRGKHGRRHGGKGHRGCRKTWAGCEQGSSMDVGQEAEPAPSGAARSQEKSGEAGGNTQNKEQDATQKQPEGTAEPMETTVKDGSAAAPNQSPSGSDDADWTLLENAGGKPSAPSQNPQEDPSYQYQTALNQMGAMGFDNEGGWLTSLLDAKGGDIVRVLDAIKIGNQPSFSGNSK

>Gallus gallus_p62_XP001233249

MAALTVKAYLLGKEDATREIRRFSLMPPVRYQAVHDRVAELFQGLLRAGPPPAFRMHYKDEDGDLIAFSSDEELDLAMPYVQDGVFRVYIKEKKECRREHRSQCSQEPPRDMVHPNVICDGCQGPVVGARFKCTVCPDYDLCSTCEGKGIHKEHNMVMFQSPLLNPFEWYPRGRWLRKMRHGVPPFPWMHCWGYPGPAAPCQNSEQAQADAAASSPPAAGEASTNSQPQDPNVTFLKNVGESVAAFLSPLGIEVDIDVEHGGQRSKVTPASPNQENNNAESSSSTLNQNSQTKPYWSNTESATVVNTVAEQIQDMVIDPVPTQMEDGSLQSQEHSESSSSSGGDEDWTHLSSKEVDPSTGELQSLQMPETDDPSSLDVAQEAPQAGPTGLREAALYPHLPPEADPRLIESLSQMLSMGFSDEGGWLTRLLQTKNCDIGAALDAIQYSKQPPSL

>Canis familiaris_p62_XP013973114

MAMSYVKDDIFRIYIKEKKECRRDHRPPCAQEAPRGLVHPNVICDGCNGPVVGTRYKCSVCPDYDLCAACEGKGLHREHSKLVFPGTFGPFSEGFSHSRWLRKLKHGHFGWPGWEMGPPGNWSPRPPRAGDARPGPAAESVSGPSEDPSVNFLKNVGESVAAALSPLGIEVDIDVEHGGKRSRLTPVSPGGSSTEDRGSSQPSSCSSDPNKPDVDRDPEGTAQALAEQMDKVALESGPPEEQMESDNCSGGDEDWTHLSSKEVDPSTGELQSLQMPESEGPGSLGSSQEGPTGLKEAALYPHLPPEADPRLIESLSQMLSAGIEVDIDVEHGGKRSRLTPVSPGGSSTEDRGSSQPSSCSSDPNKPDVDRDPEGTAQALAEQMDKVALESGPPEASTRPGPGLSLCTVSLWTPGIADPSMSWSECRWWS

**5. Lamp-1**

>*Macrobrachium rosenbergii*_Lamp-1

MAKLFAFAFALLFACGSVIGQDDTTIEPDTTLPPEPPTTVPLTTEAPITEPPATTAPPEPETTTAPETTVPPEPETTTAAPVPDTTTAPVPVTTPAPEANYNYNITENNVTCIMIEGAVTFLVNYTTVNNETKTATVVLPKHPKANSVITGTCNGTDGQEEIQVTWGPAGQSSSVKVEFGVKGDSWSVSSFVANLFMDPVTFANGTDAGKTLSLDVDYGFSPLSVPVNHSFNCHSSLSAVNITATIGGAPYSLGVSSKLDGIHIQAYNMVPYEPDFVSSVHCSADEISDVVPIAVGCALAALVVIVLIAYLVGRRRRSAAYQSV

> *Macrobrachium* nipponense_Lamp-1_ SRR496753.2184.2

MAKLFAFAFALLFACGSVIGQEDTTLEPDTTLAPTTVPPTTEAPTEPPATTTVPPEPETTTAPETTVTPEPETTTAAPVPDTTTAPAPVTTPAPEPNYNYNITENNVTCIMIEGAVTFFVNYTTVNNETKTATVVLPKHPKADSVITGTCNGTDGQEEIDVTWGSAGQSSSVKVEFGIKGDSWSVSSFVANLFMDPVTFANGTDAGKTLSLVVDYGFSPLSVPVNHSFNCHSSLSAVNITATIGDAPYTQRVSSKLEGIHIQAFNMVPYEPDFVSSVHCSADEISDVVPIAVGCALAALVVIVLIAYLVGRRRRSAAYQSV

>*Pontastacus leptodactylus*_Lamp-1_GAFS01004294

MALNFSSGVILIVLVVAGSMAMGQETDVTTDSTSMPTTRSSTSTTTSLTSTTTKPTTTTELSTAEPTTTDAPETTEPPSTSEAPTTTTEPPTTTTEMPTTETPTTPSPDTKYRVKDGNLTCIMVEGEISFAISYLTPKNDTKVAKVKVPQHEGKVSGTCNGTDGTQSITISWTDEGPSSTTFTFRKSEDNWSVSSFTASLYLNNKTFPDSLLSGKMLDLVIFSSLNHFDVAVNFSLNCHSTVSTSNVTGTLEKEEYNLPLSSSFSQIHVEAFNEIPNEPDFVASVHCAADKTSDIVPIAVGCALAGLVVIVLIAYLVGRRRRSGAYQSV

>Daphnia pulex_Lamp-1_EFX65747

MAVKKILAMLCVCICTGNALDVMASNFSDYSDQSNATTTSTIKPSTSTTTLPTPEMSTTEMSSTEMSTTPSTTPFIPTTTTSSPTTFIPVSPTYPPTPKPNPPNPAMNWVVVDEQTNVTCIVVKLAASFVIPYLKVNGQMENVTLALPSNATSSGTCNLDDLEHGQEISLQWYADKSGSPNTLTFAFHRNVSSGTEATGKESVYLSEINLSVFLDSENFVNASRVGNYSHGKLQNSLHSVPVNNSYRCNSEETFELNLIEEIAEVAIIHFAHMQTEAFRTQNDDKFHSAVDCPADKVITSDIVPIAVGCALAALVVVVLVAYLIGRRRARQRGYQSV

>Drosophila melanogaster_Lamp-1_NP610111

MFANKLLTCSALLIMLFLLSSTVFSQKLIGLPRVGKNVSDLFLEQANPLTSSSSTISTSSTTTEKPITTRSTSTITPRSTTPSTESPSTTAPVISTTIAPQPYPQPSIGAWNTSCIMLQMAAQLNFTYEAREGNFTTGLYNIPSNASVEDAECKSQTTQFIHLIWGPETSKQSLIMYFNKSNDTTVLSFMQIHLALLPEDFPDAKENQTVQLITRSDGAFKTPENMSYHCTRVQKINMTETLDAEQLIGWISVSHVQVEAFRRANDTGFSVGHDCDSSETSDVVPIAVGIALAALILVVLISYLCARRRSTSRGYMSF

>Crassostrea gigas_Lamp-1_EKC35611

MEGSFRMMVTINKTTIIVDVPSKEEVNVTGMCASGNETESSITITPKSGPLKSLTFDFKINKNKDMADKSELVSMSAEVMVDGKVEPISANKTLLVLSNPKLFYKCDVGSNFTADKVIFKYEQLKVQAFNVENEKFAENGEDCSEDVGPIPAPGNIPVNTYAVTEGNKTCIVFKGSMKFVIPYVAKSGNATEPVGLPEKFTVSGSCNFLLNGEVSQKMEVKFYEDWILTIYFTDSDNKTDVQNQLTADSSDKYYISQIELEYEYNSNLFVTADESILGKKAKFTAANLTLWETGKGKSYMCNKESTINLSSKDGFELTVSKVQYQAFKKGNSNDFGDASECAADEETNSIVPIAVGAALAGLVIIVLIAYLIGRKRSRAGYEQV

>Danio rerio_Lamp-1_NP955996

MARAAGVCWTLLMGCVFAAHAVTFEVTDGNSTCIKGELNASFSISYNTTNGTSVSVFALPASASVSERSSCGSAAVPPELALVFGDTHTHTLSLLFSRDQRLYRVSNISLQYNLSDGDIFPQSSSAGVQSVMASVSELMSARLNSTYRCVSSSSISLSAAVNLTLSGVQMEAYMSSANLSADESVCSADQPSTTVAPPPSTTTSPPPIPPVPERGNYSVTDGNGTVCVLALMGLQLNITHTTTQNQSVSELMNLQPNQTTVSGSCGVTESSLRLSDETTNLTFSFTMNSTTQKYYLSAVSVSALWPDMSVVFEAGNTSLSALQCSVGRSYVCSAQQMLSVTPVFSINTFRLQLQPFNITANRFSTAEECRVDQENMLIPIIVGAALAGLVLIVLVAYLIGRKRTHAGYQTI

>Xenopus laevis_Lamp-1_NP001087042

MSWRQVKMPVYWMAVMLLIGVVQVATAVQFEVKDGKTNITCILADLSINFSVSYNVSSKMELATFVLPSEAVTNINKSSCGVENTTAPVLAIQFGSNHSLSIHFARNNTRYEVAELVMSYNLSDKIIFPNASENGTKTVSTNKTAVLAENDTVYKCMNPHLIRMDNANATFHDIRLEAYLKQSNFSQKVSTCSEDITPTSAPAPVTTTAPVPAPVPDPPVVQYSVNRSSEPCLLAKVGLQMNITYTTKDGKNGSYVFNIESKGVTVDGNCTNTTAYLSLSTGSIDLRFNFTLNSSLEVFYLDGVSLSTGLPADANDTHFEAANSSLNYMQTNVHKSFKCNSKQTLQITDPFTVNTYHLQVQAFNSDNTFASAVECSLDENGMLVPIVVGAALAGLVLIVLIAYLIGRKRSHAGYQTI

>Rattus norvegicus_Lamp-1_NP036989

MAAPGARRPLLLLLLAGLAHSAPALFEVKDNNGTACIMASFSASFLTTYDAGHVSKVSNMTLPASAEVLKNSSSCGEKNASEPTLAITFGEGYLLKLTFTKNTTRYSVQHMYFTYNLSDTQFFPNASSKGPDTVDSTTDIKADINKTYRCVSDIRVYMKNVTIVLWDATIQAYLPSSNFSKEETRCPQDQPSPTTGPPSPSPPLVPTNPSVSKYNVTGDNGTCLLASMALQLNITYMKKDNTTVTRAFNINPSDKYSGTCGAQLVTLKVGNKSRVLELQFGMNATSSLFFLQGVQLNMTLPDAIEPTFSTSNYSLKALQASVGNSYKCNSEEHIFVSKALALNVFSVQVQAFRVESDRFGSVEECVQDGNNMLIPIAVGGALAGLVLIVLIAYLIGRKRSHAGYQTI

>*Homo sapiens*_Lamp-1_NP005552

MAAPGSARRPLLLLLLLLLLGLMHCASAAMFMVKNGNGTACIMANFSAAFSVNYDTKSGPKNMTFDLPSDATVVLNRSSCGKENTSDPSLVIAFGRGHTLTLNFTRNATRYSVQLMSFVYNLSDTHLFPNASSKEIKTVESITDIRADIDKKYRCVSGTQVHMNNVTVTLHDATIQAYLSNSSFSRGETRCEQDRPSPTTAPPAPPSPSPSPVPKSPSVDKYNVSGTNGTCLLASMGLQLNLTYERKDNTTVTRLLNINPNKTSASGSCGAHLVTLELHSEGTTVLLFQFGMNASSSRFFLQGIQLNTILPDARDPAFKAANGSLRALQATVGNSYKCNAEEHVRVTKAFSVNIFKVWVQAFKVEGGQFGSVEECLLDENSMLIPIAVGGALAGLVLIVLIAYLVGRKRSHAGYQTI
